# Classification feasibility test on multi-lead electrocardiography signals generated from single-lead electrocardiography signals

**DOI:** 10.1038/s41598-024-52216-y

**Published:** 2024-01-22

**Authors:** Gi-Won Yoon, Segyeong Joo

**Affiliations:** Department of Biomedical Engineering, Asan Medical Center, Asan Medical Institute of Convergence Science and Technology, University of Ulsan College of Medicine, Seoul, Republic of Korea

**Keywords:** Cardiology, Computer science

## Abstract

Nowadays, Electrocardiogram (ECG) signals can be measured using wearable devices, such as smart watches. Most wearable devices provide only a few details; however, they have the advantage of recording data in real time. In this study, 12-lead ECG signals were generated from lead I and their feasibility was tested to obtain more details. The 12-lead ECG signals were generated using a U-net-based generative adversarial network (GAN) that was trained on ECG data obtained from the Asan Medical Center. Subsequently, unseen PTB-XL PhysioNet data were used to produce real 12-lead ECG signals for classification. The generated and real 12-lead ECG signals were then compared using a ResNet classification model; and the normal, atrial fibrillation (A-fib), left bundle branch block (LBBB), right bundle branch block (RBBB), left ventricular hypertrophy (LVH), and right ventricular hypertrophy (RVH) were classified. The mean precision, recall, and f1-score for the real 12-lead ECG signals are 0.70, 0.72, and 0.70, and that for the generated 12-lead ECG signals are 0.82, 0.80, and 0.81, respectively. In our study, according to the result generated 12-lead ECG signals performed better than real 12-lead ECG.

## Introduction

Cardiovascular diseases (CVDs) comprise a series of heart blood-vessel abnormalities, which are one of leading reasons for deaths worldwide^1^. ECG signals are typically used in the early prediction and general diagnosis of abnormal heart rhythms. ECG signals are used to measure the heart’s electrical activity and rhythm through methods, such as resting ECG and Holter monitoring, that are conventional ECG measurement methods. Typically, 12-lead ECG signals are used to diagnose cardiac diseases^2^. Heart diseases often cause an irregularity in the heart called arrhythmia, wherein A-fib is the most common cardiac arrhythmia. However, real-time ECG measurement is required for early diagnoses.

In this regard, wearable ECG measurement devices are currently in use, with more being developed. Holter ECG devices were developed for long-term ECG monitoring^3^; however, owing to their limitations such as patient discomfort and limited mobility, ECG monitoring devices, such as patches and watches, were developed. These methods can be used to only measure one of the 12 leads. It is now possible to monitor the patient’s ECG from their home. These ECG monitoring devices are less complex and expensive compared to conventional methods. However, wearable devices, such as patches and smart watches, have a critical limitation; they cannot measure synchronous multiple leads^4^. Generally, lead I can be representative of limb leads but not precordial leads. Therefore, abnormal cardiac diseases, such as RBBB, LBBB, RVH, and LVH, cannot be diagnosed^5^. Although single-lead devices are widely used, they are rarely used for diagnosing all cardiac diseases. Due to their lack of information and difficulties mainly A-fib is currently diagnosed by single lead ECG devie^6^.

Because ECG signals represent a vector of the heart, automated ECG-based classification models have become popular. Most classification models employ 12-lead ECG signals or only lead II signal for learning^7^. Various deep learning methods, from convolutional neural networks (CNNs) to the more recent transformer encoder models, have been implemented in ECG classification problems and produced promising results^8–10^. Moreover, data generation models, such as the GANs^11^, transformers^12^, and diffusion models^13^, have been implemented on ECG signals for data synthesis^14,15^.

For related works, there have been attempts to classify A-fib using single-lead ECG signals and the data augmentation method^16,17^. Hatamian et al.^18^ proposed a method of classifying A-fib by combining the data augmentation and deep learning methods. However, the GAN input in this method is noises, and the type of GAN used is unconditional GAN; therefore, the generated data does not represent other lead vectors. Moreover, precordial leads were not considered. Lan et al.^19^ used short-time Fourier transform and GAN to classify and augment data. However, related classification studies mainly focused on augmentation to increase the amount of data. These methods have been verified to increase the performance accuracy; however, the augmented data were not verified.

There have been several studies on lead conversion. Sohn et al. proposed a method for reconstructing a 12-lead ECG from a 3-lead patch device employing an LSTM network^20^. However, their work requires a device measuring at least three leads, whereas our work only needs lead I. Therefore, it is limited in application to single-lead measurement devices. Afrin et al. proposed a handheld ECG device measuring single-lead ECG, which could measure lead I, lead II, and lead III^21^. Three different leads are measured asynchronously. Upon the previously measured ECG history, 12-lead ECGs are synchronously reconstructed. Therefore, the proposed method additionally needs previously measured ECG for reconstruction. Huang et al. proposed an ECG system reconstruction method from temporally asynchronous bipolar ECG recordings^22^. Their reconstruction algorithm is based on multiple ECGs recorded asynchronously from different sites. However, when deriving the optimal weight coefficient, only 11 subjects were recorded, and external data were not tested. SynSigGAN is typically used to generate biomedical signals^23^ and implement discrete wavelet transformation and Bidirectional Long Short-Term Memory (Bi-LSTM)^24^ layers for the generation model. However, inputs are typically treated as latent variables; therefore, their use is limited to data augmentation. Shin et al.^25^ proposed a method for generating a photoplethysmography (PPG) signal from an ECG signal using an LSTM and a CNN. Lee et al.^26^ proposed the R-peak alignment and time sequence embedding method to transform a one-dimensional time series into a two-dimensional time-series for enhancing the performance of GANs on two-dimensional time series. Chest leads were also converted from limb leads with high accuracy. However, the R-peak, which was used as the median value, resulted in more than one beat during data preprocessing and the model inputs were lead II. Existing methods related to lead conversion performed well. However, the generated ECG signals in these studies were not evaluated based on a classification comparison with real ECG signals. Therefore, the frechet distance (FD) and mean squared error (MSE) scores should be used as evaluation scores, and a comparison with real ECG signals should be considered alongside a Turing test to achieve the usefulness of generated ECG.

In this study, all 12-lead ECG signals were generated using lead I, and their feasibility for usage was determined through a classification performance test. Our study's novelty lies in the analysis of ECG signals generated from Lead I through classification tests, demonstrating their feasibility. In this study, the possibility of generating ECG signals for diagnostic implementation, which reinforces the limitation of single-lead ECG measurement devices, was presented. The proposed method can be applied in out-of-hospital ECG monitoring care without using multiple lead measurement devices.

## Methods

**Figure 1:**
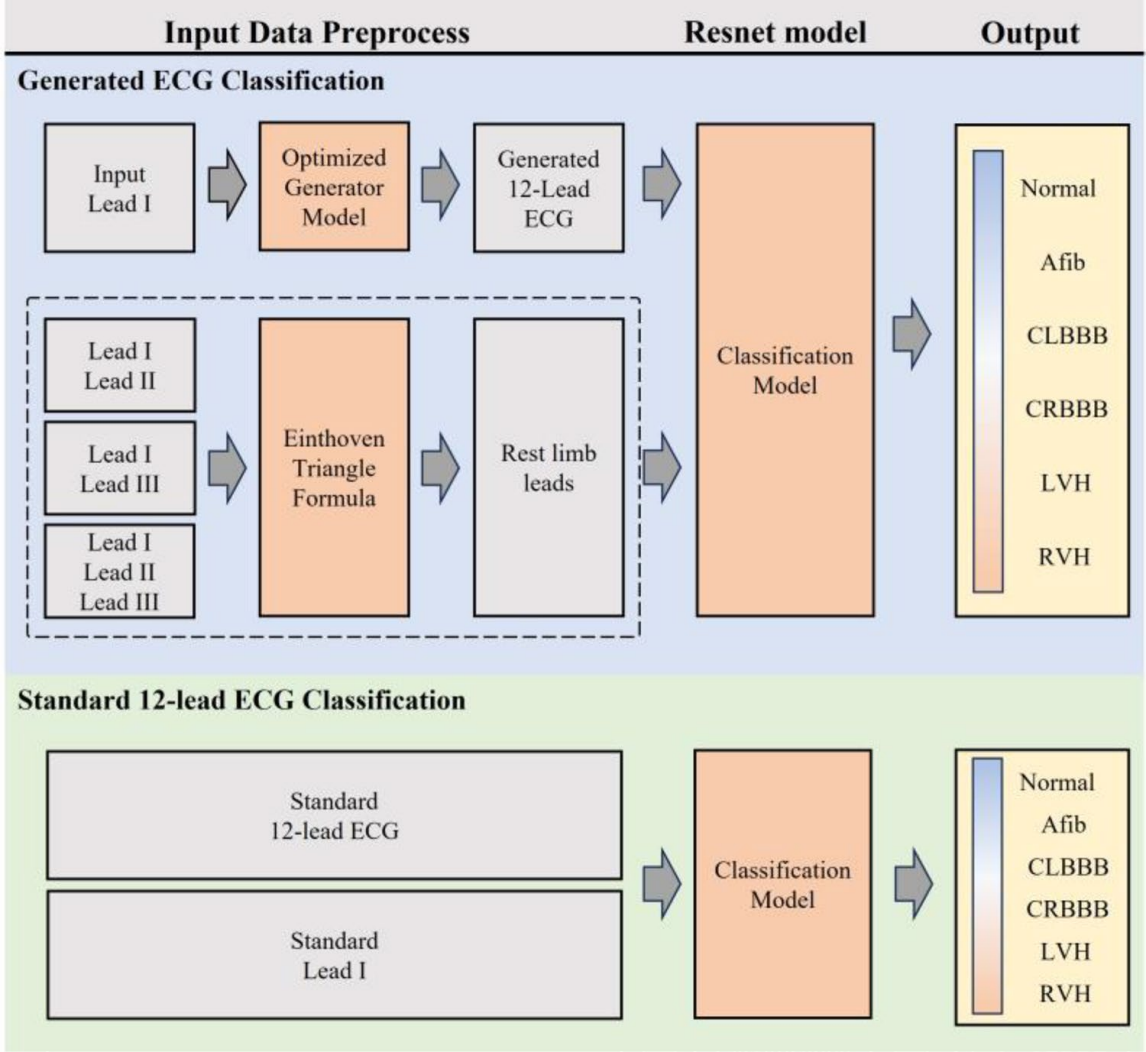
Overview of the proposed method. Generated ECG and real ECG signals are equally preprocessed, trained, and classified using the same ResNet model. The output of the classification model is normal, A-fib, CLBBB, CRBBB, LVH, and RVH.

**Figure 2:**
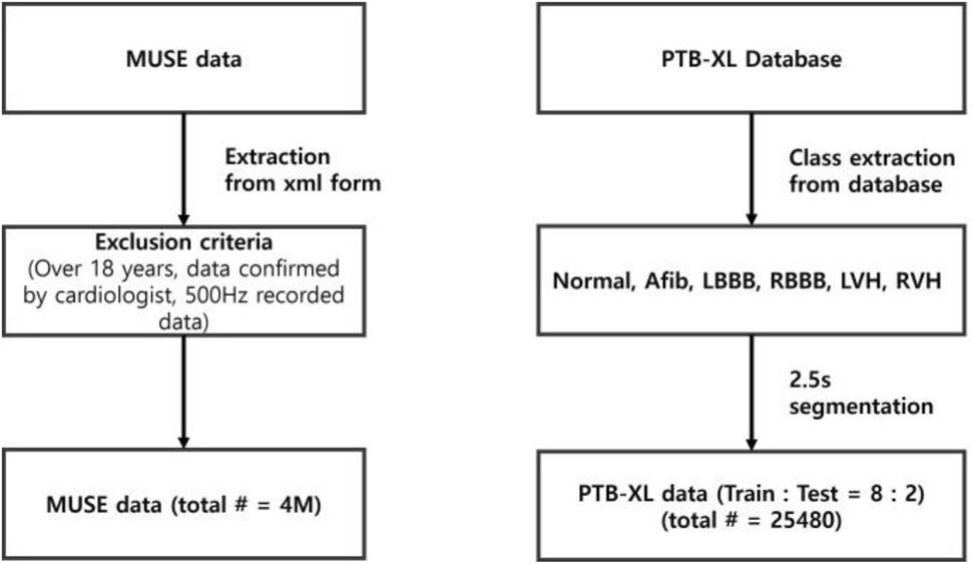
Data exclusion and preprocessing. For the MUSE data, data on patients under 18 years old, unconfirmed data, and patient records with sampling rates under 500 Hz were excluded. Both databases were segmented into 2.5 s segments for training.

In this study, the ECG generation model was based on that in our previous study^27^. The pix2pix GAN model was trained using MUSE data on patients who had visited the Seoul Asan Medical Center Hospital between January 01, 2001, and February 28, 2022. For classification, the PTB-XL database was used as external data. As illustrated in Fig. 2, six classes were extracted from the PTB-XL database for the classification test. Evaluation was based on the F1-score, precision, recall, and accuracy. The overview of this study is illustrated in Fig. 1.

### Datasets and preprocessing

The 12-lead ECG data used in this study were obtained from the MUSE and PTB-XL databases^28^. The PTB-XL dataset contains 21,837 records obtained from 18,885 patients, and the MUSE database comprises 4 million records obtained from the Asan Medical Center Hospital. The experimental protocols in the data were approved by the Institutional Review Board (IRB) at the Asan Medical Center Hospital, under the approval number IRB No. 2022–0781. All methods were carried out in accordance with relevant guidelines and regulations and informed consent was obtained from all subjects and/or their legal guardian(s).

The duration of both datasets was 10 s and the sampling rate was 500 Hz, wherein the number of sampling points were 5,000. For the generation model, lead I ECG signals obtained from the MUSE database were considered as the input ECG signals, and the remaining leads as reference signals. Additionally, all 12-lead ECG signals from the PTB-XL dataset were used in the classification model. The data descriptions are listed in Supplementary Tables S1, S2 and S3. To generate and classify raw ECG signals using our model, none of the conventional methods, such as filtering or adjusting the baseline, were implemented. Figure 2 illustrates the data preprocess and exclusion criteria. For the MUSE database records, data on patients under 18 years old, unconfirmed data and patient records with sampling rates under 500 Hz were excluded. Furthermore, the 10 s records were segmented into 2.5 s intervals, each containing 1250 sampling points.

### GAN architecture

GAN consists of two main networks: a generator and discriminator^11^. The basis of GAN is a minimax game between the generator and discriminator. In this study, the generator considers lead I as the input and synthesizes the remaining leads; and the discriminator distinguishes the generated signals from the real ones. Figure 1 depicts the overall architecture of the proposed model. The proposed model follows the main objective of conditional GAN, which can be expressed as shown in (1). Conditional GANs^29^ learn mapping based on the relationship between the signal x and random noise vectors z and y^14^.

1 $${\mathcal{L}}_{cGAN} \left( {G,\;D} \right) = {\mathbb{E}}_{x,y} \left[ {logD\left( {x,\;y} \right)} \right] + {\mathbb{E}}_{x,z} [\log \left( {1 - D\left( {x,\;G\left( {x,\;z} \right)} \right)} \right],$$

2 $${\mathcal{L}}_{L1} \left( G \right) = {\mathbb{E}}_{x,y,z} \left[ {y - G\left( {x,\;z} \right)} \right],$$

where G tries to minimize the objective of GAN against D, which tries to maximize it (1). Moreover, L1 loss was used (2); thus, the final objective of GAN was represented as follows:

3 $$G^{*} = arg\mathop {\min }\limits_{G} \mathop {\max }\limits_{D} {\mathcal{L}}_{cGAN} \left( {G,\;D} \right) + \lambda {\mathcal{L}}_{L1} \left( G \right).$$

### GAN and discriminator network

The generator in this study comprises a U-net based encoder-decoder. The U-net generator is depicted in Supplementary Figure S6. The encoder consists of seven convolution layers, with batch normalization and Leaky ReLU applied in all except the first layer. The decoder is composed of seven up-convolution layers. For all Leaky ReLU functions, the slope was set to 0.2; the kernel size and stride length were 4 and 2, respectively. The discriminator, depicted in Supplementary Figure S7, contains five convolution layers with batch normalization and Leaky ReLU. A convolution layer is added after the last layer to map to a one-dimensional output, followed by a Sigmoid function. The slope for all Leaky ReLU functions is 0.2, with a kernel size of four and a stride length of two. The learning rate is set to 0.0005 for the generator and 0.0001 for the discriminator. Additionally, Adam is employed for hyperparameter optimization, and the batch size is set to 32. A total of 11 models were trained to generate 11 leads. As mentioned in the 'Datasets and Preprocessing' section, the input and output dimensions of the generator, and the input of the discriminator, are all (batch size, 1, 1250).

### Evaluation method

The classification of the generated 12-lead ECG signals was performed using the ResNet model. The normal ECG, RBBB, LBBB, LVH, RVH, and A-fib values were then used to evaluate the classification classes. The A-fib and normal ECG were used because most out-of-hospital wearable devices are used to detect AF, and both normal ECG and AF can be classified using single-lead ECG measurement^9^. By contrast, RBBB, LBBB, LVH, and RVH were diagnosed using the precordial leads (V1, V2, V3, V4, V5, and V6). To test the feasibility of the 12-lead ECG generated from lead I, 5 different methods were compared. First, the classification results of the generated 12-lead ECG and real lead-I ECG signals were compared. To verify the disadvantages of single-lead measurement, the classification performances of the generated 12-lead ECG and real lead-I were compared. Second, the classification results of the generated 12-lead and real 12-lead ECG signals was compared. From the Einthoven triangle formula^30^ (4–7) was then applied to the generated 12-lead ECG signals to conduct the ablation study. Two different limb leads are required when applying the Einthoven triangle formula. Therefore, three different groups of leads were used in the experiment: input lead I and generated lead II, input lead I and generated lead III, and input lead I and generated lead II, III. The groups were separately evaluated to determine the best outcome results and differences in the number of generated leads.

4 $$Lead \;III = Lead\; II - Lead\; I$$

5 $$Lead\; aVR = - \left( {Lead\; I + Lead \;II} \right)/2$$

6 $$Lead \;aVL = Lead\; I - \left( {Lead \;II} \right)/2$$

7 $$Lead \;aVF = Lead \;II - \left( {Lead\; I} \right)/2$$

All the five different sets of methods were evaluated based on their precision, recall and f1-score values (10–12); and PTB-XL external data were used to train and evaluate each classification method.

8 $$Precision = \frac{True\; positive}{{True \;positive + False\; positive}}$$

9 $$Recall = \frac{True \;positive}{{True \;positive + False \;negative}}$$

10 $$F1\; score = \frac{2}{{\frac{1}{Precision} + \frac{1}{Recall}}}$$

## Results

**Figure 3:**
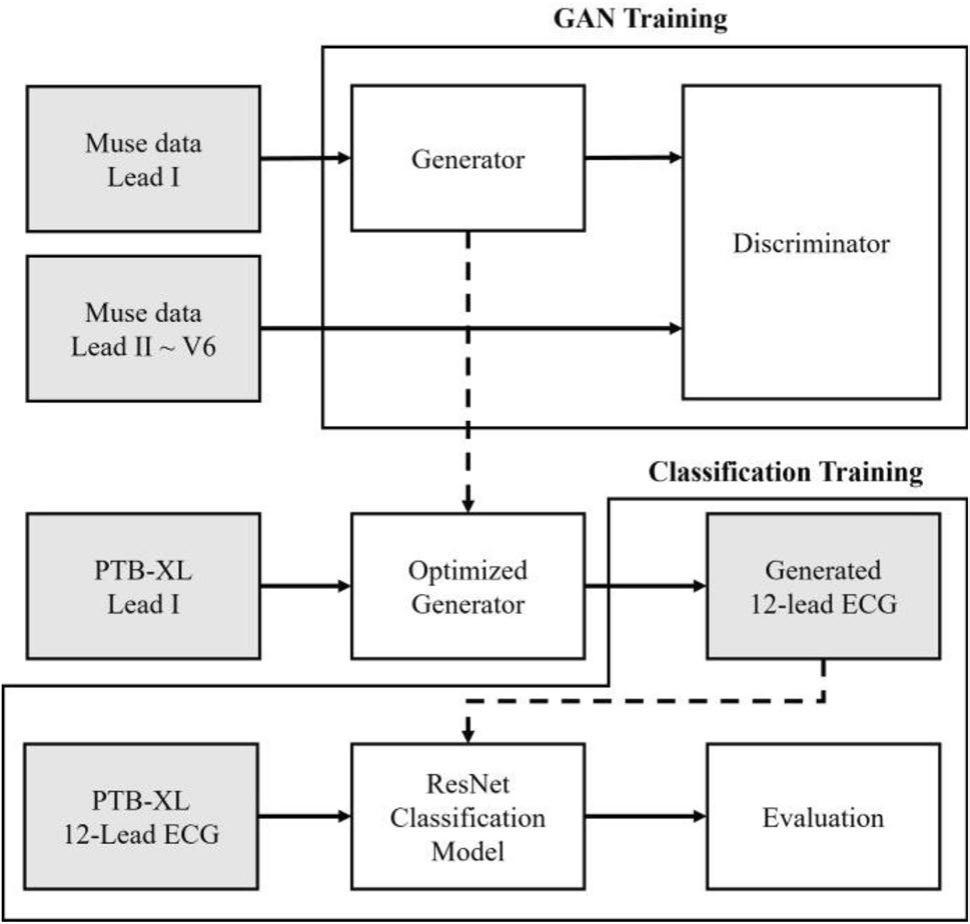
Overview of the model training method. Generator models were trained on MUSE data, and the optimized model was implemented to generate 12-lead ECG signals for training. Additionally, the ResNet model was used for classification.

In this section, the generated signals from the PTB-XL database and their evaluation scores are presented and compared. Figure 3 illustrates the generation and classification processes using lead I. The evaluation of the generation model performance was performed in our previous study^27^. The evaluation scores for all five results is shown in Table 1 and Table 2.

**Table 1:** Evaluation of the performance score of the generated ECG signals.

	Generated 12-Lead			Lead II by GAN			Lead III by GAN			Lead II, III by GAN		
	Precision	Recall	F1-score	Precision	Recall	F1-score	Precision	Recall	F1-score	Precision	Recall	F1-score
Normal	**0.89**	**0.92**	**0.91**	0.88	0.82	0.85	**0.92**	0.81	0.86	0.79	0.93	0.85
Afib	**0.96**	0.76	0.84	0.94	0.72	0.81	0.92	0.88	**0.9**	0.85	**0.91**	0.88
CLBBB	**1**	**0.96**	**0.98**	1	0.95	0.97	1	0.94	0.97	1	0.92	0.96
CRBBB	0.87	0.77	0.82	0.89	0.78	0.83	**0.89**	**0.85**	**0.87**	0.84	0.81	0.83
LVH	**0.82**	**0.94**	**0.87**	0.77	0.96	0.85	0.81	0.93	0.87	0.91	0.80	0.85
RVH	0.38	0.47	0.42	0.49	0.31	0.38	**0.52**	**0.51**	**0.51**	0.42	0.19	0.26

Significant are in value [bold].

**Table 2:** Evaluation of the Performance Score of Real ECG signals.

	Real Lead I			Real 12-Lead ECG		
	Precision	Recall	F1-score	Precision	Recall	F1-score
Normal	0.63	0.77	0.69	0.72	0.84	0.78
Afib	0.66	0.69	0.67	0.70	0.88	0.78
CLBBB	0.78	0.73	0.75	0.81	0.85	0.83
CRBBB	0.64	0.71	0.67	0.70	0.71	0.70
LVH	0.68	0.56	0.62	0.87	0.61	0.72
RVH	0.25	0.16	0.20	0.40	0.43	0.41

The precision, recall, and f1-score values of the generated.

12-lead ECG signals and classification performance results of real lead-I are shown in Table 1, where the best results are highlighted in bold. The generated 12-lead ECG signals exhibited the best results followed by the generated lead II. This shows that multi-lead ECG classification is more accurate. Particularly, the classification results of the abnormal.

ECG signals that are typically diagnosed at the precordial lead show a significant difference. The classification performance of all real 12-leads is shown in Table 2, where real lead exhibited poor results using both the 12-lead ECG and single lead I signals.

**Figure 4:**
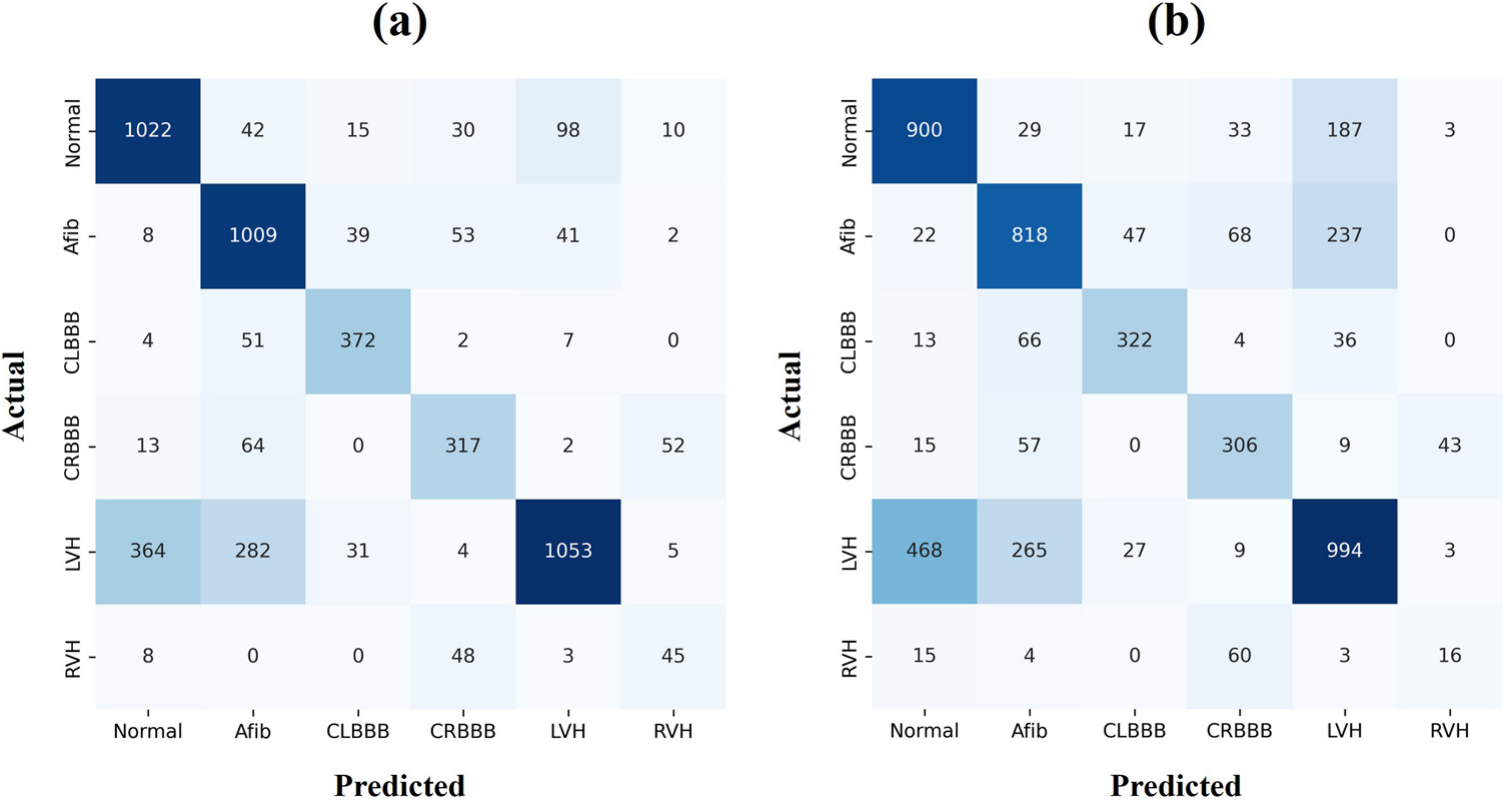
Confusion matrix for the real ECG. (**a**) 12-lead ECG signal results, and (**b**) classification results using lead I.

**Figure 5:**
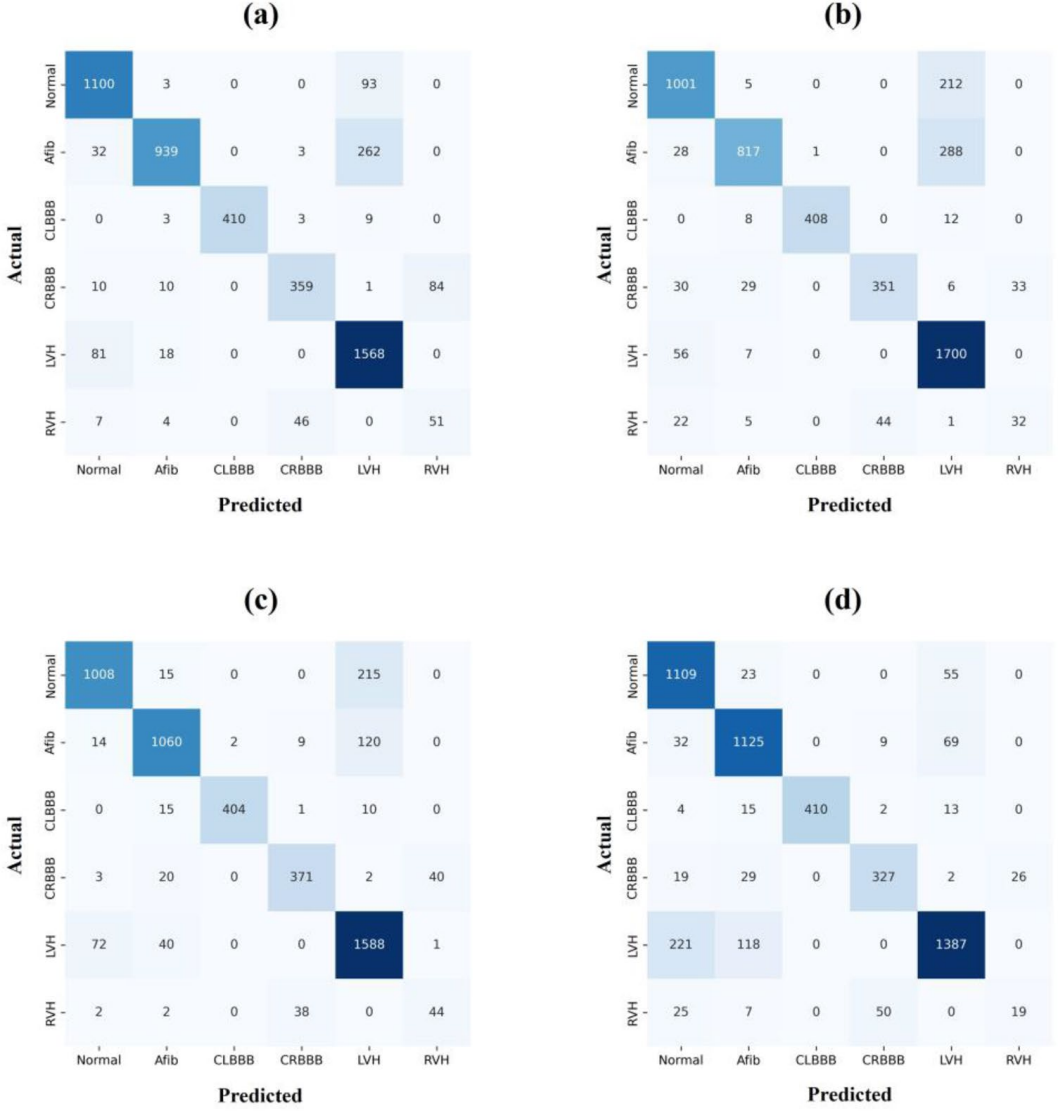
Confusion matrix for the generated ECG. (**a**) Generated 12-lead ECG signal results, and (**b**), (**c**), and (**d**) are the results of the Einthoven lead conversion. In (**b**), only lead II was generated using GAN and limb lead and the rest were calculated using the Einthoven formula. In (**c**), only lead III was generated using GAN and limb lead and the rest were calculated using the Einthoven formula. In (**d**), lead II and lead III were generated using GAN and limb lead and the rest were calculated using the Einthoven formula.

The confusion matrix results are depicted in Fig. 4. Additionally, the experimented results of the three different lead groups are listed in Table 1 and the confusion matrix is presented in Fig. 5. No significant differences in the results can be observed when the Einthoven formula is used to calculate the other limb leads. Moreover, the ROC and AUC results illustrated in Supplementary Figure S1 exhibit no significant difference. Therefore, generating only Lead II or Lead III and calculating the rest of limb leads using the Einthoven formula reduces both the model complexity and time. The generated 12-lead ECG and reference ECG are illustrated in Supplementary Figure S2. The capability of the generated ECG signals in diagnoses was also tested to verify the classification results of the real ECG signals.

## Discussion

This study demonstrates that generated ECG signals are capable of diagnosing CVDs. Table 3 shows previous studies related to ECG generation by GANs and have evaluated their work with classifications performance that was listed in review paper by Laurenz Berger^15^. However, as shown in Table 3 most of the previous works’ purpose was focused mainly on solving imbalanced data problems. Also, the input data were noise and simulator where our study focused on lead-to-lead conversion. Previous and related studies have only focused on data augmentations and generated signals were not from single lead ECG. A detailed example of the generated ECG signals is shown in Supplementary Figure S1.

**Table 3:** Comparison with previous ECG Generation model studies.

Study	Purpose	Generated ECG implementation	GAN type	Input
Golany et al.^31^	Augmentation for imbalanced data	Train stage	Unconditional GAN	Noise
Wang et al.^32^	Augmentation for imbalanced data	Train stage	Conditional GAN	Simulator
Golany et al.^33^	Augmentation for imbalanced data	Train stage	ODE GAN	Simulator
Shaker et al.^34^	Augmentation for imbalanced data	Train stage	Unconditional GAN	Noise
Rath et al.^35^	Augmentation for imbalanced data	Train stage	Conditional GAN	Real ECG
Ma et al.^36^	Augmentation for imbalanced data	Train/Test stage	Unconditional GAN	Noise
Hatamian et al.^18^	Augmentation for imbalanced data	Train/Test stage	Unconditional GAN	Noise
Wang et al.^37^	Augmentation for imbalanced data	Train/Test stage	Unconditional GAN	Noise
Thambawita et al.^38^	Augmentation for imbalanced data	Test stage	Unconditional GAN	Noise
Zhang et al.^17^	Augmentation for imbalanced data	Test stage	Conditional GAN	Real ECG
Yang et al.^39^	Augmentation for imbalanced data	Train/Test stage	Unconditional GAN	Noise
Brophy et al.^40^	Augmentation for imbalanced data	Train/Test stage	Unconditional GAN	Noise
Zhou et al.^41^	Augmentation for imbalanced data	Train/Test stage	Unconditional GAN	Noise
Li et al.^42^	Augmentation for imbalanced data	Train/Test stage	Unconditional GAN	Noise
Ours	Lead to lead conversion	Test stage	Conditional GAN	Real Lead I

Single-lead ECG signals can be can better classified by implementing the proposed method to classify CVDs, which improves the disadvantages of single-lead ECG signals. This method enables the real-time analysis of ECG signals through single-lead ECG measurement, thereby allowing the use of single-lead ECG measurement devices, such as smart watches, on both patients and the general public. Therefore, the proposed method can be used to alert users and patients of potential danger. Additionally, single-lead measurement, which is a more comfortable method, can be adopted in hospitals instead of 12-lead standard ECG measurement.

The results of the RVH performance were lower than those of the other classes. Moreover, the F1-score difference was up to 0.49 lower than that of the normal class, mainly owing to the low quantity of the RVH data in the PTB-XL database used to train the classification model compared to that used in the other classes. However, its performance was still higher than that of real 12-lead ECG signal classification. 12-lead ECG signals were generated from lead I rather than lead II because general single-lead ECG devices comprise mainly smart watches, which measure lead I.

The classification experiments were performed using an external dataset that was not used to train the generation model. A comparison of the classification of the generated 12-lead and real lead I signals was also performed. This comparison was performed to determine whether the generated ECG signals will have a better classification performance than real lead I. However, the results were more dramatic in precordial-based CVD diagnosis. This result shows that single-lead ECG measurement devices are not capable of diagnosing the various CVD types; however, applying the proposed method improves their classification capability.

Standard ECG signals are used in 12-lead ECG measurement. Multiple electrodes are attached to the surface of the patients’ body, which makes it hard to obtain ECG signals in the long term. However, various devices have been developed with the growth of the single-lead ECG device market. Owing to their real-life ECG measurement capabilities, they are used to detect cardiac diseases, such as A-fib. However, these single-lead measurement devices generally detect or diagnose cardiac diseases based on the rhythmic features; therefore, it is nearly impossible to detect diseases that are diagnosed based on the amplitude or via comparison with other leads.

This study presented the feasibility of generated ECG signals for use in diagnosis. The obtained results were better than those of real ECG signals, which can be implemented in single lead devices. The accuracies, precisions, and F1 scores of the generated 12-lead ECG are shown in Tables 1 and 2. The normal class values are 0.89, 0.92, and 0.91; the A-fib class values are 0.96, 0,76, and 0.84; the LBBB values are 1, 0.96, and 0.98; the RBBB results are 0.87, 0.77, and 0.82; the LVH results are 0.82, 0.94, and 0.87; and the RVH values are 0.38, 0.47, and 0.42, respectively. Among the six classes, A-fib was the only class that was not in sinus rhythm, resulting in higher performance.

The proposed method can also be used to provide insights into various pathological cardiac diagnoses features. This will allow the monitoring of personalized ECG signals during in- and out-of-hospital care, where the cardiologist keeps patient records over a long time. Moreover, further assessment can be made by the cardiologist when a remarkable CVD is detected during the patient’s daily life.

Most of all, the novelty of our study is:

1. A large dataset of over 400 million data is used to train the generative model.
2. No other study has investigated the use of generated ECG signals for diagnosis.
3. Generated ECG classification exhibits a better performance than reference single-lead ECG classification, indicating that the information obtained from the precordial leads are crucial.

As shown in Tables 1 and 2, the proposed method produces a better performance than real ECG classification. Several questions need to be addressed regarding why the generated ECG signal classification results show higher performance. Our proposed model has been trained on a dataset of 4 million samples and has the capability to generate ECG signals that closely resemble real ones. The primary difference between the generated ECG signals and the corresponding reference ECG signals is that the generated ones can fill in missing data and reduce baseline wandering problems shown in Supplementary Figure S4 and S5. However, the crucial outcome of the study is that CVD diagnosis using the entire 12-lead ECG performs better when employing our proposed method.

However, a few limitations exist in this study. First, 6 CVD types containing both precordial and limb leads were classified. Nonetheless, there are various of types of CVDs, such as acute MI (AMI), that are life-threatening. Certain MI, such as ST elevation, are fairly classified using DL (deep learning)^43–45^. However, there are very few AMI record data available owing to its high mortality rate. In the future, more focus should be placed on critical CVDs, which can require out-of-hospital care. Second, for the lead I ECG signals, the input in the proposed method was based on standard 12-lead ECG records. No open data were measured using both the single-lead device and standard 12-lead ECG. However, a few single-lead ECG signals were generated from smart watches, as depicted in Supplementary figure S3, and classified to show the concept of our method. It was important for the proof of concept to show that the ECG signals obtained from single-lead devices can be used to generate 12-lead ECG signals and to detect CVDs.

## Conclusion

This study presents a method for generating 12-lead ECG signals that can be used to classify CVDs using DL. ECG data obtained from the Asan medical center and containing 400 million records was used. External data from the PTB-XL database were also used to classify 6 types of cardiac diseases present in the limb and precordial leads. Additionally, the performance of the classification results was compared with those of real and generated ECGs. Consequently, the proposed method exhibited outstanding results during classification, which can be applied in real-life ECG monitoring. Single-lead ECG devices are simple and comfortable to wear; however, owing to the lack of lead information, rhythm features are mainly used to detect abnormal ECG. This approach can be used to solve for the disadvantages of single-lead ECG devices, thereby helping in out-of-hospital CVD detection, which is a crucial step in personalized medicine.

### Supplementary Information

Supplementary Information.

## Data Availability

The datasets generated or analyzed during the current study are not publicly available due to the restriction policy of the hospital but are available from the corresponding author upon a reasonable request. PTB-XL data base are public data which is available at https://physionet.org/content/ptb-xl/1.0.3/.

## Code availability

All algorithms used in this study are available by contacting the corresponding author at any time.
