# Supplementary material for: Classification feasibility test on multi-lead electrocardiography signals generated from single-lead electrocardiography signals
Source: Sci Rep. 2024 Jan 22;14:1888. doi: 10.1038/s41598-024-52216-y (PMC10803292; doi:10.1038/s41598-024-52216-y)
Supplement: Supplementary file 1 — Supplementary Information. [file 41598_2024_52216_MOESM1_ESM.docx]

**Supplementary Material**

Classification Feasibility Test of Generated Multi-Lead Electrocardiography from Single-Lead Electrocardiography

Gi-Won Yoon^a,1^, Segyeong Joo^a*^

^a^Department of Biomedical Engineering, Asan Medical Institute of Convergence Science and Technology, Asan Medical Center, University of Ulsan College of Medicine, Seoul, Republic of Korea.

# Abstract

Conventional ECG measurement methods are resting ECG, Holter monitoring, and treadmill method. Unlike conventional ECG measurement methods, now days ECG signals can be measured by wearable devices such as smart watches. Most wearable devices provide fewer details, however, has advantage of recording data in real life. To overcome the limitation of having fewer details 12-lead ECG were generated from lead I and the feasibility were tested in this study. 12-lead ECG were generated by U-net based GAN. U-net based GAN were trained by ECG data obtained from Asan Medical Center. Unseen data from PTB-XL PhysioNet data were used to generate 12-lead ECG and for classification. Generated 12-lead ECG and real 12-lead ECG were compared by ResNet classification model. Normal, Afib, CLBBB, CRBBB, LVH and RVH were classified. Afib were chosen to compare limb lead performance and others were chosen to compare the precordial lead performance. Precision, recall and f1-score were calculated to compare classification performance between generated and real ECG data. Mean precision, recall and f1-score for real 12-lead ECG were 0.70, 0.72 and 0.70, respectively. Mean precision, recall and f1-score for generated 12-lead ECG were 0.82, 0.80 and 0.81, respectively. Generated 12-lead ECG score resulted higher performance than real 12-lead ECG. In this study we have evaluated our 12-lead generative model by classifying 6 diagnostic classes. The results show that generated 12-lead ECG can be used to diagnose cardiac diseases.

# **Muse database description**

Muse data obtained from Asan Medical Center Hospital contains over 400 million records. Records under 18 years old, unconfirmed data, and sampling rate under 500Hz have been excluded. Table 1 shows Muse Acronym and its number of records.

Supplementary Table S1

| **Code** | **MUSE Acronym** | **Text** | **# records** |
| --- | --- | --- | --- |
| 1 | SNF | STATEMENT NOT FOUND | 1212 |
| 2 | PEDANL | ** * Pediatric ECG analysis * ** | 3630 |
| 3 | AGSPAMI | *** Age and gender specific ECG analysis *** | 19424 |
| 4 | $ACS | ** Acute Cardiac Syndrome criteria ** | 3277 |
| 5 | DICTATION | Report dictated, transcription pending | 5771 |
| 6 | $TWLVW | Leads V2, V3, V4, and V6 are interpolated | 18430 |
| 8 | $VLDFMT | Waveform is valid only when viewed in 4x2.5 format with lead II as the rhythm lead | 87348 |
| 9 | $5SECLD1 | Only the first 5 seconds of lead I are valid | 5460 |
| 10 | $RDBC1 | Reserved for Database Conversion | 5765 |
| 11 | $RDBC2 | Reserved for Database Conversion | 5765 |
| 12 | $RDBC3 | Reserved for Database Conversion | 5765 |
| 13 | $SERREM | The system removed serial comparison statements because | 3635 |
| 14 | $NOT12SL | this ECG was not analyzed with 12SL | 73960 |
| 15 | $NOT12SL2 | the 1st previous ECG was not analyzed with 12SL | 75170 |
| 16 | $NOT12SL3 | this patient has a test analyzed with the HEART algorithm | 79719 |
| 19 | SRTH | Sinus rhythm | 213710 |
| 20 | ARAT | (Atrial rate= | 638 |
| 21 | SBRAD | Sinus bradycardia | 402623 |
| 22 | NSR | Normal sinus rhythm | 1756560 |
| 23 | STACH | Sinus tachycardia | 131400 |
| 24 | MSBRAD | Marked sinus bradycardia | 48870 |
| 25 | RABRAD | Low right atrial bradycardia | 1903 |
| 26 | RATACH | Low right atrial tachycardia | 1873 |
| 27 | LABRAD | Left atrial bradycardia | 1274 |
| 28 | LATACH | Left atrial tachycardia | 1265 |
| 29 | RAR | Low right atrial rhythm | 2302 |
| 30 | LAR | Left atrial rhythm | 1622 |
| 31 | NOPF | (no P-waves found) | 1380 |
| 32 | BLKED | blocked | 2 |
| 33 | ACCEL | Accelerated | 8455 |
| 34 | JUNBRAD | Junctional bradycardia | 2855 |
| 41 | JBRAD | Unusual P axis and short PR, probable junctional bradycardia | 23038 |
| 42 | JR | Unusual P axis and short PR, probable junctional rhythm | 23409 |
| 43 | JTACH | Unusual P axis and short PR, probable junctional tachycardia | 23361 |
| 61 | EABRAD | Unusual P axis, possible ectopic atrial bradycardia | 6167 |
| 62 | EAR | Unusual P axis, possible ectopic atrial rhythm | 10447 |
| 63 | EATACH | Unusual P axis, possible ectopic atrial tachycardia | 4427 |
| 64 | EARO | Ectopic atrial rhythm | 2357 |
| 100 | PRINT | PR interval | 3986 |
| 101 | FAV | with 1st degree AV block | 72751 |
| 102 | SPR | with short PR | 93067 |
| 103 | MBZI | with 2nd degree AV block (Mobitz I) | 74026 |
| 104 | MBZII | with 2nd degree AV block (Mobitz II) | 74026 |
| 105 | SAV | with 2nd degree AV block | 72752 |
| 106 | CHB | with complete heart block | 73201 |
| 107 | VAVB | with variable AV block | 72148 |
| 108 | AVDIS | with AV dissociation | 71553 |
| 111 | SABII | with 2nd degree SA block (Mobitz II) | 74041 |
| 112 | SABI | with 2nd degree SA block (Mobitz I) | 74066 |
| 113 | PAUSE | with sinus pause | 71544 |
| 141 | W2T1 | with 2:1 AV conduction | 72147 |
| 142 | W3T1 | with 3:1 AV conduction | 72150 |
| 143 | W4T1 | with 4:1 AV conduction | 72147 |
| 144 | W5T1 | with 5:1 AV conduction | 72165 |
| 161 | AFIB | Atrial fibrillation | 172664 |
| 162 | FLUT | Atrial flutter | 23826 |
| 163 | CRS | Coarse | 1 |
| 164 | ATAC | Atrial tachycardia | 663 |
| 171 | RVR | with rapid ventricular response | 103312 |
| 172 | SVR | with slow ventricular response | 91969 |
| 173 | ABER2 | with premature ventricular or aberrantly conducted complexes | 103347 |
| 174 | CJP | with a competing junctional pacemaker | 80535 |
| 175 | IRREG | with undetermined rhythm irregularity | 72674 |
| 176 | IRR | Irregular | 180 |
| 177 | $SWITH | with | 70308 |
| 178 | $SOR | or | 3947 |
| 179 | $SAND | and | 14171 |
| 181 | ABER | with premature ventricular or aberrantly conducted complexes | 103347 |
| 183 | APCX | atrial-paced complexes | 783 |
| 184 | VPCX | ventricular-paced complexes | 2036 |
| 185 | AVPCX | AV dual-paced complexes | 1339 |
| 186 | ASVPCX | atrial-sensed ventricular-paced complexes | 2641 |
| 187 | SCX | sinus complexes | 653 |
| 188 | SVCX | supraventricular complexes | 698 |
| 189 | INTRIN | intrinsic complexes | 636 |
| 190 | PROAV | with prolonged AV conduction | 74746 |
| 211 | OCC | with occasional | 116489 |
| 212 | FREQ | with frequent | 90441 |
| 221 | PSVC | premature supraventricular complexes | 1304 |
| 222 | PAC | premature atrial complexes | 1217 |
| 223 | PJC | premature junctional complexes | 1216 |
| 231 | PVC | premature ventricular complexes | 1217 |
| 232 | PVCF | premature ventricular and fusion complexes | 16596 |
| 233 | CSEC | and consecutive | 16299 |
| 234 | BIGEM | in a pattern of bigeminy | 7609 |
| 235 | WQTACH | Wide QRS tachycardia | 2650 |
| 236 | NQTACH | Narrow QRS tachycardia | 1621 |
| 237 | $SWQR | Wide QRS rhythm | 6933 |
| 238 | $SIVR | Idioventricular rhythm | 1367 |
| 241 | PEC | premature ectopic complexes | 1216 |
| 242 | JESC | with junctional escape complexes | 72790 |
| 243 | VESC | with ventricular escape complexes | 72650 |
| 244 | $SFUS | fusion complexes | 606 |
| 245 | $SRETC | with retrograde conduction | 72332 |
| 246 | $SABCOND | aberrant conduction | 610 |
| 247 | $SCAPTUR | sinus/atrial capture | 612 |
| 248 | $SVTACH | Ventricular tachycardia | 1580 |
| 249 | $SVFIB | Ventricular fibrillation | 609 |
| 251 | SAR | with sinus arrhythmia | 135681 |
| 252 | MSAR | with marked sinus arrhythmia | 86235 |
| 265 | PR-SBRAD | Probable sinus bradycardia, verify AV conduction | 3073 |
| 266 | SUP-TACH | Supraventricular tachycardia | 4158 |
| 267 | JUNCT-R | Junctional rhythm | 16325 |
| 268 | IDIO-R | Idioventricular rhythm with AV block | 73516 |
| 269 | VENT-RTH | Ventricular rhythm | 610 |
| 270 | J-TACH | Junctional tachycardia | 631 |
| 271 | SVT | Supraventricular tachycardia | 4158 |
| 272 | VTACH | Ventricular tachycardia (ventricular or supraventricular with aberration) | 78961 |
| 273 | AFL | Atrial flutter | 23826 |
| 274 | VENT-FUS | with ventricular fusion | 71543 |
| 275 | J-ESC | with junctional escape | 71543 |
| 276 | ESCBT | with escape beat | 71543 |
| 277 | TVT | with transient ventricular tachycardia | 72171 |
| 278 | WEKH | with Mobitz I (Wenckebach) block | 72822 |
| 279 | PO-ATP | Possible wandering atrial pacemaker | 166827 |
| 280 | MULT-AT | Multifocal atrial tachycardia | 1244 |
| 281 | COMP-HB | Complete heart block | 1212 |
| 282 | AV-COND | Suspect AV conduction defect | 1821 |
| 283 | AB-VENT | with intermittent aberrant ventricular conduction | 72763 |
| 284 | SA-BLK | with SA block or transient AV block | 77912 |
| 285 | SAB | with sinus arrest or transient AV block | 77910 |
| 287 | LHR | Low heart rate, verify AV conduction | 3033 |
| 288 | AFL-BL | Atrial flutter with 2 to 1 block | 97188 |
| 289 | BIVPCK | Biventricular pacemaker detected | 1676 |
| 290 | PCK | Electronic ventricular pacemaker | 17137 |
| 291 | DPCK | Demand pacemaker, interpretation is based on intrinsic rhythm | 18421 |
| 292 | APCK | Electronic atrial pacemaker | 6544 |
| 293 | AVPCK | AV sequential or dual chamber electronic pacemaker | 11020 |
| 294 | EDP | Electronic demand pacing | 15779 |
| 295 | APR | Atrial-paced rhythm | 3733 |
| 296 | VPR | Ventricular-paced rhythm | 3580 |
| 297 | ASVPR | Atrial-sensed ventricular-paced rhythm | 5766 |
| 298 | AVDPR | AV dual-paced rhythm | 3015 |
| 299 | UR | Undetermined rhythm | 12797 |
| 300 | WPWA | Ventricular pre-excitation, WPW pattern type A | 3933 |
| 302 | WPWB | Ventricular pre-excitation, WPW pattern type B | 3813 |
| 303 | ALTWPW | with fusion or intermittent ventricular pre-excitation (WPW) | 77980 |
| 304 | WPW | Wolff-Parkinson-White | 3063 |
| 305 | CWRT | Clockwise rotation of the heart, may invalidate criteria for ventricular hypertrophy | 10017 |
| 306 | CCWRT | Counterclockwise rotation of the heart, may invalidate criteria for ventricular hypertrophy | 10015 |
| 307 | DXTRO | Dextrocardia | 508 |
| 320 | CUR-UND | Current undetermined rhythm precludes rhythm comparison, needs review | 5460 |
| 321 | PRV-UND | Previous ECG has undetermined rhythm, needs review | 4647 |
| 322 | VENT-RAT | Vent. rate | 13879 |
| 323 | RHY | Rhythm | 2 |
| 324 | PRM-CON | The premature contractions | 1215 |
| 325 | CONSEC | Consecutive | 3 |
| 326 | WITH-DEM | with a demand pacemaker | 86320 |
| 327 | BASIC | Basic rhythm | 608 |
| 350 | RAE | Right atrial enlargement | 16738 |
| 360 | LAE | Left atrial enlargement | 77456 |
| 369 | BAE | Biatrial enlargement | 5393 |
| 370 | LAD | Leftward axis | 668 |
| 371 | ALAD | Abnormal left axis deviation | 5774 |
| 372 | LAD3 | Left axis deviation | 121311 |
| 380 | RAD | Rightward axis | 67761 |
| 381 | ARAD | Abnormal right axis deviation | 5780 |
| 382 | RSAD | Abnormal right superior axis deviation | 10325 |
| 383 | RAD4 | Right axis deviation | 19400 |
| 384 | RAD5 | Right superior axis deviation | 12088 |
| 390 | INDAX | Indeterminate axis | 3620 |
| 391 | NWA | Northwest axis | 5216 |
| 395 | AXIS | QRS axis | 2204 |
| 396 | SHFT-LFT | shifted left | 604 |
| 397 | SHFT-RGT | shifted right | 605 |
| 410 | LOWV | Low voltage QRS | 41597 |
| 411 | PULD | Pulmonary disease pattern | 14729 |
| 412 | S1S2S3 | S1-S2-S3 pattern, consider pulmonary disease, RVH, or normal variant | 12773 |
| 440 | RBBB | Right bundle branch block | 114878 |
| 441 | RVE+ | , plus right ventricular hypertrophy | 4561 |
| 442 | RBBRVH | Right bundle branch block -or- Right ventricular hypertrophy | 162503 |
| 445 | IRBBB | Incomplete right bundle branch block | 64697 |
| 446 | IRB-RVE | . | 33 |
| 450 | RSR | RSR' or QR pattern in V1 suggests right ventricular conduction delay | 15730 |
| 451 | SRSRO | RSR' pattern in V1 | 2055 |
| 460 | LBBB | Left bundle branch block | 22810 |
| 465 | ILBBB | Incomplete left bundle branch block | 6681 |
| 470 | AFB | Left anterior fascicular block | 32034 |
| 471 | PFB | Left posterior fascicular block | 12323 |
| 478 | BIFB1 | (RBBB and left anterior fascicular block) | 21290 |
| 479 | BIFB2 | (RBBB and left posterior fascicular block) | 21261 |
| 480 | BIFB | *** Bifascicular block *** | 12192 |
| 481 | TRIFB | Trifascicular block | 615 |
| 482 | IVCB | Nonspecific intraventricular block | 1224 |
| 487 | IVCD | Nonspecific intraventricular conduction delay | 1834 |
| 520 | RVH | Right ventricular hypertrophy | 42469 |
| 521 | RVH-2ST | Right ventricular hypertrophy with repolarization abnormality | 168764 |
| 540 | LVH | Voltage criteria for left ventricular hypertrophy | 105889 |
| 541 | LVH2 | Left ventricular hypertrophy | 105760 |
| 542 | QRSV | Minimal voltage criteria for LVH, may be normal variant | 165245 |
| 543 | QRSW | with QRS widening | 89574 |
| 544 | 2ST | with repolarization abnormality | 125688 |
| 545 | QRSW-2ST | with QRS widening and repolarization abnormality | 118356 |
| 548 | LVH3 | Moderate voltage criteria for LVH, may be normal variant | 78499 |
| 570 | BIVH | Biventricular hypertrophy | 10712 |
| 571 | PMDPV | Prominent mid-precordial voltage, | 7115 |
| 572 | QV6 | Deep Q wave in lead V6, | 3040 |
| 573 | PPV | Prominent posterior voltage | 5160 |
| 574 | PLV | Prominent lateral voltage | 1212 |
| 575 | QIII | Deep Q in lead III | 2420 |
| 700 | SMI | Septal infarct | 33040 |
| 701 | SMI-LAE | . | 33 |
| 740 | AMI | Anterior infarct | 30632 |
| 760 | LMI | Lateral infarct | 20087 |
| 780 | IMI | Inferior infarct | 125824 |
| 782 | MAFB | (masked by fascicular block?) | 2780 |
| 795 | RVI | with right ventricular involvement | 72151 |
| 800 | PXT | , with posterior extension | 76098 |
| 801 | IPMI | Inferior-posterior infarct | 13740 |
| 802 | POSTMI | Posterior infarct | 5074 |
| 803 | QESPMI | Increased R/S ratio in V1, consider early transition or posterior infarct | 18628 |
| 805 | RV4R | Inferior injury pattern suggests right ventricular involvement, recommend adding leads V3r and V4r to confirm | 27752 |
| 806 | CRVI | Consider right ventricular involvement in acute inferior infarct | 12744 |
| 810 | ASMI | Anteroseptal infarct | 24394 |
| 820 | ALMI | Anterolateral infarct | 14338 |
| 821 | STEMI | ** ** ACUTE MI / STEMI ** ** | 10805 |
| 822 | NSTEMI | ** ** ACUTE MI / non-STEMI ** ** | 4239 |
| 823 | LBBBNEW | ** ** Consider ACUTE MI if LBBB is new ** ** | 6051 |
| 826 | LBBBACS | ** ** LBBB with primary ST-T abnormality - Consider ACUTE CORONARY SYNDROME (ACS) ** ** | 82832 |
| 827 | LBBBAMI | ** ** LBBB with primary ST elevation abnormality - PROBABLE ACUTE MI ** ** | 112384 |
| 828 | AIS | ** ** Consider ACUTE CORONARY SYNDROME (ACS) ** ** | 4909 |
| 829 | ACUMI | ** ** ACUTE MI ** ** | 3025 |
| 830 | AC | , possibly acute | 10478 |
| 831 | AU | , age undetermined | 198656 |
| 832 | OLD | , old | 610 |
| 833 | NEW | , new | 838 |
| 840 | INC-MI | Increased evidence of infarction in | 2427 |
| 841 | DEC-MI | Questionable change in initial forces of | 9218 |
| 842 | QUE-INICHG | Questionable change in initial forces of | 9218 |
| 843 | CRI-FOR | Criteria for | 5782 |
| 844 | CITED | (cited on or before | 23659 |
| 845 | MINI-CRIT | Minimal criteria for | 5958 |
| 846 | BORD-CRIT | Borderline criteria for | 24006 |
| 880 | MISIZ | *** QRS contour suggests infarct size is probably | 4244 |
| 881 | VSMA | very small | 607 |
| 882 | SMA | small | 0 |
| 883 | MOD | moderate | 1 |
| 884 | LARG | large | 1 |
| 885 | VLAR | very large | 608 |
| 900 | NST | Nonspecific ST abnormality | 78888 |
| 901 | PCARD | Acute pericarditis | 2158 |
| 902 | SERYR1 | ST elevation, consider early repolarization, pericarditis, or injury | 64184 |
| 903 | SERYR2 | ST elevation, probably due to early repolarization | 33886 |
| 904 | NSTE | Nonspecific ST elevation | 31457 |
| 920 | SINJ | Septal injury pattern | 1217 |
| 930 | AINJ | Anterior injury pattern | 6445 |
| 940 | LINJ | Lateral injury pattern | 2710 |
| 950 | IINJ | Inferior injury pattern | 6308 |
| 960 | ASINJ | Anteroseptal injury pattern | 1217 |
| 961 | ALINJ | Anterolateral injury pattern | 1687 |
| 962 | ILINJ | Inferolateral injury pattern | 1403 |
| 963 | IIOHAI | ST elevation, consider inferior injury or acute infarct | 42375 |
| 964 | AIOHAI | ST elevation, consider anterior injury or acute infarct | 42373 |
| 965 | LIOHAI | ST elevation, consider lateral injury or acute infarct | 38424 |
| 966 | ALIHAI | ST elevation, consider anterolateral injury or acute infarct | 38425 |
| 967 | ILIHAI | ST elevation, consider inferolateral injury or acute infarct | 38426 |
| 968 | INJONV | ST elevation, consider injury or variant associated with LVH | 109376 |
| 1000 | REPOL | Early repolarization | 25920 |
| 1001 | JSTN | Junctional ST depression, probably normal | 39412 |
| 1002 | JST | Junctional ST depression, probably abnormal | 36813 |
| 1020 | STDIG | ST abnormality, possible digitalis effect | 44159 |
| 1021 | NST2 | Nonspecific ST abnormality | 78888 |
| 1022 | STDEP | ST depression, consider subendocardial injury or digitalis effect | 60036 |
| 1023 | NSTD | Nonspecific ST depression | 31435 |
| 1024 | STDEP2 | ST depression, consider subendocardial injury | 33007 |
| 1040 | SSBINJ | Marked ST abnormality, possible septal subendocardial injury | 38318 |
| 1050 | ASBINJ | Marked ST abnormality, possible anterior subendocardial injury | 43043 |
| 1060 | LSBINJ | Marked ST abnormality, possible lateral subendocardial injury | 40888 |
| 1070 | ISBINJ | Marked ST abnormality, possible inferior subendocardial injury | 44723 |
| 1071 | MSTDIL | Marked ST abnormality, possible inferolateral subendocardial injury | 38467 |
| 1080 | MSTDAS | Marked ST abnormality, possible anteroseptal subendocardial injury | 38188 |
| 1081 | MSTDAL | Marked ST abnormality, possible anterolateral subendocardial injury | 39307 |
| 1082 | STDPIN | ST depression in | 31772 |
| 1083 | STELIN | ST elevation in | 32163 |
| 1084 | WSTR | with strain pattern | 73107 |
| 1100 | ST& | ST & | 30816 |
| 1104 | ST-NOLDEP | ST no longer depressed in | 34770 |
| 1105 | ST-LESDEP | ST less depressed in | 32352 |
| 1106 | ST-MORDEP | ST more depressed in | 36285 |
| 1107 | ST-NOWDEP | ST now depressed in | 34241 |
| 1108 | ST-DEPREP | ST depression has replaced ST elevation in | 78821 |
| 1115 | QUE-STCHG | Questionable change in ST segment | 33776 |
| 1116 | ST-(INC) | Non-specific change in ST segment in | 35058 |
| 1117 | ST-(DEC) | Non-specific change in ST segment in | 35058 |
| 1120 | ST-MORELV | ST more elevated in | 36175 |
| 1121 | ST-LESELV | ST less elevated in | 32256 |
| 1122 | ST-ELVPRS | ST elevation now present in | 34247 |
| 1123 | ST-NOLELV | ST no longer elevated in | 33908 |
| 1124 | ST-ELVREP | ST elevation has replaced ST depression in | 78733 |
| 1138 | STABAND | ST abnormality and | 50746 |
| 1139 | SNDQA | , may be secondary to QRS abnormality | 13225 |
| 1140 | NT | Nonspecific T wave abnormality | 123578 |
| 1141 | NSTT | Nonspecific ST and T wave abnormality | 94615 |
| 1142 | QRST | Abnormal QRS-T angle, consider primary T wave abnormality | 26521 |
| 1143 | LNGQT | Prolonged QT | 155236 |
| 1144 | BOQTI | Borderline QT interval | 19342 |
| 1145 | ILT | T wave abnormality, consider inferolateral ischemia | 21127 |
| 1150 | AT | T wave abnormality, consider anterior ischemia | 45488 |
| 1151 | MAT | Marked T wave abnormality, consider anterior ischemia | 46917 |
| 1160 | LT | T wave abnormality, consider lateral ischemia | 75414 |
| 1161 | MLT | Marked T wave abnormality, consider lateral ischemia | 78360 |
| 1170 | IT | T wave abnormality, consider inferior ischemia | 46504 |
| 1171 | MIT | Marked T wave abnormality, consider inferior ischemia | 47397 |
| 1172 | MILT | Marked T wave abnormality, consider inferolateral ischemia | 21732 |
| 1180 | ALT | T wave abnormality, consider anterolateral ischemia | 43573 |
| 1181 | MALT | Marked T wave abnormality, consider anterolateral ischemia | 54371 |
| 1182 | TINVIN | T wave inversion in | 1833 |
| 1200 | T-WAVE | T waves | 605 |
| 1201 | T-INC | T wave amplitude has increased in | 14057 |
| 1203 | T-DEC | T wave amplitude has decreased in | 12274 |
| 1207 | LOWT-INVT | Flat T waves have replaced inverted T waves in | 4842 |
| 1208 | QUE-TCHG | Questionable change in T waves | 3564 |
| 1210 | LOWT-NOL | Flat T waves no longer evident in | 3628 |
| 1211 | LESS-FLTT | Fewer leads exhibit flat T waves in | 3628 |
| 1212 | LOWT-NOW | Flat T waves now evident in | 3023 |
| 1213 | MORE-FLTT | More leads exhibit flat T waves in | 7574 |
| 1214 | NSTNL | Nonspecific T wave abnormality no longer evident in | 130283 |
| 1215 | NSTNW | Nonspecific T wave abnormality now evident in | 129448 |
| 1216 | NSTLS | Nonspecific T wave abnormality, improved in | 126002 |
| 1217 | NSTMR | Nonspecific T wave abnormality, worse in | 129746 |
| 1218 | NSTFT | Nonspecific T wave abnormality has replaced inverted T waves in | 142499 |
| 1219 | NSTNF | Inverted T waves have replaced nonspecific T wave abnormality in | 11362 |
| 1220 | T-INVNOW | T wave inversion now evident in | 9029 |
| 1221 | T-INVMOR | T wave inversion more evident in | 8878 |
| 1222 | INVT-LOWT | Inverted T waves have replaced flat T waves in | 4842 |
| 1223 | T-LESINV | T wave inversion less evident in | 5096 |
| 1224 | T-INVNOL | T wave inversion no longer evident in | 8911 |
| 1250 | QT-LONG | QT has lengthened | 7213 |
| 1251 | QT-SHRT | QT has shortened | 8778 |
| 1252 | RAT-DEC | Although rate has decreased | 9575 |
| 1253 | RAT-INC | Although rate has increased | 11946 |
| 1254 | WITH-RATINC | with rate increase | 71543 |
| 1255 | WITH-RATDEC | with rate decrease | 71542 |
| 1300 | NO-SERIAL | No previous ECGs available | 82574 |
| 1301 | COMPAR | When compared with ECG of | 244522 |
| 1302 | POOR-DAT | Poor data quality in current ECG precludes serial comparison | 8870 |
| 1303 | NO-SERCMP | Serial comparison not performed, all previous tracings are of poor data quality | 14557 |
| 1304 | DEMOGR | Warning: demographic data different | 1825 |
| 1305 | NO-CHG | No significant change was found | 100216 |
| 1306 | SUNCNF | (Unconfirmed) | 69 |
| 1340 | CRIT | *** Critical Test Result: | 1818 |
| 1342 | CVHIHR | High HR | 605 |
| 1343 | CVLOHR | Low HR | 605 |
| 1346 | CVLQT | Long QTc | 607 |
| 1360 | CVSTEMI | STEMI | 3 |
| 1361 | CVACS | ACS / Ischemia | 1218 |
| 1362 | CVAVB | AV Block | 606 |
| 1363 | CVARRHY | Arrhythmia | 3 |
| 1400 | AND | and | 14171 |
| 1401 | HOWEVER | however | 2 |
| 1402 | HWV-IT | however it | 606 |
| 1403 | LFREQ | Less frequent | 608 |
| 1404 | MFREQ | More frequent | 4555 |
| 1405 | NOLONG | is no longer | 3874 |
| 1406 | NOW | is now | 5545 |
| 1407 | HAS-CHG | has changed | 606 |
| 1408 | HAS-NOTCHG | has not changed | 1210 |
| 1409 | ARE-NOW | are now | 7932 |
| 1410 | PRESENT | present | 1 |
| 1411 | HAV-NOTCHG | have not changed | 1211 |
| 1412 | HAV-CHG | have changed | 607 |
| 1415 | HAS-REP | has replaced | 14632 |
| 1416 | HAS-INC | has increased | 10737 |
| 1417 | HAS-DEC | has decreased | 8366 |
| 1418 | ARE-NOL | are no longer | 8949 |
| 1419 | QRS | QRS | 5 |
| 1420 | QRS-DUR | QRS duration | 2512 |
| 1421 | QRS-VOL | QRS voltage | 808 |
| 1422 | QUE-CHG | Questionable change in | 2353 |
| 1423 | ACUT | Acute | 229 |
| 1424 | EVO | Serial changes of evolving | 3092 |
| 1425 | SERCHG | Serial changes of | 2130 |
| 1426 | SNGCH | Significant changes have occurred | 3024 |
| 1427 | DTOFF | Manual comparison required, data off line and on volume | 19014 |
| 1428 | ANACP | Manual comparison required for analog tracing | 6977 |
| 1430 | NOPHONE | Manual comparison required, cannot contact main system | 3643 |
| 1450 | SEP | Septal leads | 933 |
| 1451 | ANT | Anterior leads | 18549 |
| 1452 | LAT | Lateral leads | 14311 |
| 1453 | INF | Inferior leads | 21241 |
| 1454 | POS | Posterior leads | 4558 |
| 1455 | ANTSEP | Anteroseptal leads | 756 |
| 1456 | ANTLAT | Anterolateral leads | 5450 |
| 1457 | INFPOS | Inferoposterior leads | 4555 |
| 1458 | IFLAT | Inferolateral leads | 1202 |
| 1459 | RECP | reciprocal | 6 |
| 1460 | ACSBCAUS | ECG interpretation of ACS is based on presence of symptoms and | 20221 |
| 1462 | CROACS | ECG not diagnostic for Acute Coronary Syndrome; consider clinical findings | 13590 |
| 1500 | POOR | Poor data quality | 5230 |
| 1501 | POWER | Powerline interference | 612 |
| 1502 | BASELINE | Baseline wander | 14805 |
| 1503 | MUSCLE | Muscle tremor | 4554 |
| 1504 | ELECTR | Electrode noise | 608 |
| 1505 | DISC | disconnected | 4 |
| 1510 | LEAD | in lead | 607 |
| 1511 | LEADS | in leads | 607 |
| 1537 | EL-NAP | NAP | 0 |
| 1538 | EL-NST | NST | 2 |
| 1539 | EL-NAX | NAX | 0 |
| 1540 | EL-RA | RA | 1 |
| 1541 | EL-LA | LA | 0 |
| 1542 | EL-RL | RL | 1 |
| 1543 | EL-LL | LL | 0 |
| 1544 | LD-LIMB | Limb lead | 604 |
| 1545 | EL-H | H | 0 |
| 1546 | EL-E | E | 1 |
| 1547 | EL-I | I | 0 |
| 1548 | EL-M | M | 0 |
| 1550 | LD-I | I | 0 |
| 1551 | LD-II | II | 0 |
| 1552 | LD-V1 | V1 | 2 |
| 1553 | LD-V2 | V2 | 0 |
| 1554 | LD-V3 | V3 | 3 |
| 1555 | LD-V4 | V4 | 0 |
| 1556 | LD-V5 | V5 | 18 |
| 1557 | LD-V6 | V6 | 12 |
| 1558 | LD-V7 | V7 | 2 |
| 1559 | LD-V8 | V8 | 0 |
| 1560 | LD-V9 | V9 | 0 |
| 1562 | LD-V2R | V2r | 1 |
| 1563 | LD-V3R | V3r | 4 |
| 1564 | LD-V4R | V4r | 1 |
| 1565 | LD-V5R | V5r | 19 |
| 1566 | LD-V6R | V6r | 13 |
| 1567 | LD-V7R | V7r | 3 |
| 1568 | LD-V8R | V8r | 1 |
| 1569 | LD-V9R | V9r | 1 |
| 1570 | LD-A1 | A1 | 0 |
| 1571 | LD-A2 | A2 | 0 |
| 1572 | LD-A3 | A3 | 3 |
| 1573 | LD-A4 | A4 | 0 |
| 1574 | LD-III | III | 0 |
| 1575 | LD-AVR | aVR | 1 |
| 1576 | LD-AVL | aVL | 0 |
| 1577 | LD-AVF | aVF | 0 |
| 1578 | LD-MVR | mVR | 1 |
| 1579 | LD-D | D | 0 |
| 1580 | LD-A | A | 0 |
| 1581 | LD-J | J | 0 |
| 1582 | LD-X | X | 0 |
| 1583 | LD-Y | Y | 0 |
| 1584 | LD-Z | Z | 0 |
| 1585 | LD-MY | mY | 0 |
| 1586 | LD-MZ | mZ | 0 |
| 1587 | LD-CC5 | CC5 | 18 |
| 1588 | LD-CM5 | CM5 | 18 |
| 1601 | LD-R | R | 1 |
| 1602 | LD-L | L | 0 |
| 1603 | LD-N | N | 0 |
| 1604 | LD-F | F | 0 |
| 1605 | LD-C1 | C1 | 0 |
| 1606 | LD-C2 | C2 | 0 |
| 1607 | LD-C3 | C3 | 3 |
| 1608 | LD-C4 | C4 | 0 |
| 1609 | LD-C5 | C5 | 18 |
| 1610 | LD-C6 | C6 | 11 |
| 1611 | LD-C7 | C7 | 2 |
| 1612 | LD-C8 | C8 | 0 |
| 1613 | LD-C9 | C9 | 0 |
| 1615 | LD-C2R | C2r | 1 |
| 1616 | LD-C3R | C3r | 4 |
| 1617 | LD-C4R | C4r | 1 |
| 1618 | LD-C5R | C5r | 19 |
| 1619 | LD-C6R | C6r | 12 |
| 1620 | LD-C7R | C7r | 3 |
| 1621 | LD-C8R | C8r | 1 |
| 1622 | LD-C9R | C9r | 1 |
| 1669 | PMFAIL | *** Suspect unspecified pacemaker failure | 3060 |
| 1670 | PDIG | , probably digitalis effect | 20759 |
| 1671 | ODIG | or digitalis effect | 25285 |
| 1672 | ARM | *** Suspect arm lead reversal, interpretation assumes no reversal | 10101 |
| 1673 | QCERR | *** Poor data quality, interpretation may be adversely affected | 69384 |
| 1674 | AHE | Acquisition hardware fault prevents reliable analysis, carefully check ECG record before interpreting | 14651 |
| 1675 | MRR | Manual reading required due to inconsistent morphologies | 7578 |
| 1676 | $SANLERR3 | ** Less than 4 QRS complexes detected, no interpretation possible ** | 6141 |
| 1677 | $SANLERR1 | *** Memory allocation failure, no ECG interpretation possible *** | 8876 |
| 1678 | $SANLERR2 | ** No QRS complexes found, no ECG analysis possible ** | 5534 |
| 1679 | NSTDLDS | ** Nonstandard lead placement, ECG interpretation not available ** | 19033 |
| 1680 | PO | Possible | 150775 |
| 1682 | CRO | Cannot rule out | 42028 |
| 1683 | COMMA | , | 4 |
| 1684 | NML | Normal ECG | 1034193 |
| 1687 | ABR | Otherwise normal ECG | 337654 |
| 1693 | BORDE | Borderline ECG | 276703 |
| 1694 | BO | Borderline | 18096 |
| 1699 | AB | Abnormal ECG | 1086974 |

# PTB-XL Database Description

Supplementary Table S2

PTB-XL Data Description

| Clinical Characteristics | PTB-XL database | Description |
| --- | --- | --- |
| Age (years) | 59.83 ± 16.95 |  |
| Gender (male: female) | 11379 : 10458 |  |
| Weight (Kg) | 70.99 ± 15.97 |  |
| Height (cm) | 166.71 ± 10.86 |  |
| **Diagnostic statement** |  |  |
| NORM | 9528 | Normal ECG |
| STTC | 5788 | ST/T-Change |
| HYP | 2819 | Hypertrophy |
| MI | 6886 | Myocardial Infarcation |
| CD | 5772 | Conduction Disturbance |
| **Rhythm statement** |  |  |
| SR | 16782 | Sinus Rhythm |
| AFIB | 1514 | Atrial Fibrillation |
| ST | 826 | Sinus Tachycardia |
| SA | 772 | Sinus Arrhythmia |
| SB | 637 | Sinus Bradycardia |
| PACE | 296 | Normal Functioning Artificial Pacemaker |
| SVA | 157 | Supraventricular Arrhythmia |
| BIGU | 82 | Bigeminal Pattern |
| AF | 73 | Atrial Flutter |
| SVT | 27 | Supraventricular Tachycardia |
| PSVT | 24 | Paroxysmal Supraventricular Tachycardia |
| TRIGU | 20 | Trigeminal Pattern |
|  |  |  |

Supplementary Table S3

Distribution of 6 Class

| Diagnostic | Muse # records (prevalence %) | | | PTB-XL # records (prevalence %) |
| --- | --- | --- | --- | --- |
| Normal | 1034193 (5.81) | | | 9528 (66.24) |
| AFIB | 172664 (0.97) | | | 1514 (10.53) |
| CLBBB | 22810 (0.13) | | | 536 (3.73) |
| CRBBB | 114878 (0.65) | | | 542 (3.77) |
| LVH | 105889 (0.60) | | | 2137 (14.86) |
| RVH | 42469 (0.24) | | | 126 (0.88) |
|  |  |  |  |  |

# ROC and AUC Results of Generated ECG Classification Result

For more detailed results on generated 12-Lead ECG classification results ROC figure are shown in Fig.1. Also generated 12-Lead ECG examples are shown in Fig.2.


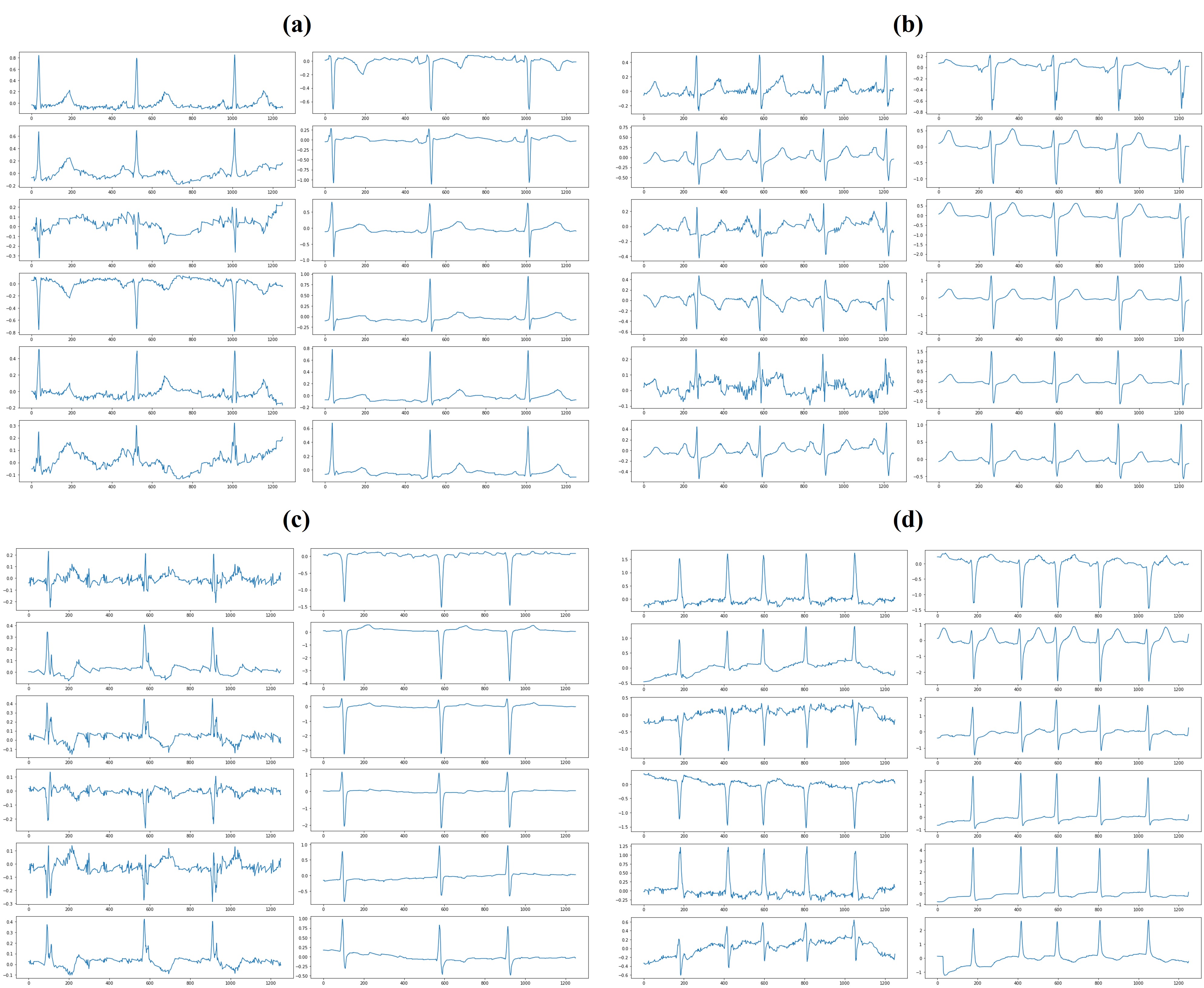


**Supplementary Figure S2 | Generated ECG from PTB-XL database**. (a) and (b) are example of generated Normal ECG signal, (c) and (d) are example of generated Afib ECG. Left column of each figure are limb leads Lead I, Lead II, Lead III, Lead aVR, Lead aVL, Lead aVF and right column is precordial leads from V1 to V6.


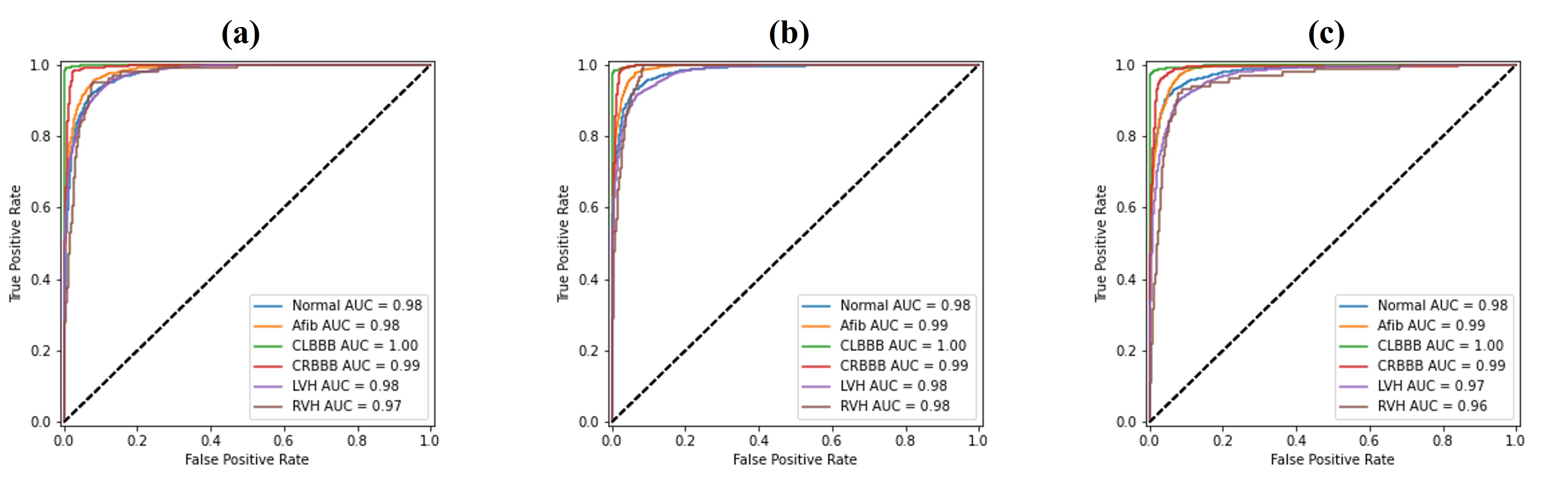


**Supplementary Figure S1 | ROC and AUC results of Einthoven experiment**. In (a) only lead II was generated in limb lead and rest were calculated by Einthoven formula, in (b) only lead III was generated limb lead rest were calculated by Einthoven, in (c) lead II and lead III were generated in limb lead rest were calculated by Einthoven.

# Single Lead ECG Device Test

For feasibility of our work we have tested our method on apple watch. Apple watch are capable of measuring ECG signal which we can obtain its raw data. The basic data given by the watch are username, user information, recorded date, sampling rate, and ECG data. The sampling rate recorded by the watch is 512Hz and the unit of ECG is $\mu V$. Therefore we have resampled the ECG data to 500Hz before usage. Fig.3. shows example of ECG obtained from the watch. Top left ECG signal indicates the ECG measured from the watch. First column are generated ECG Lead II, Lead III, Lead aVR, Lead aVL, and Lead aVF by our proposed method. The right column are generated precordial leads which are V1, V2, V3, V4, V5, and V6.


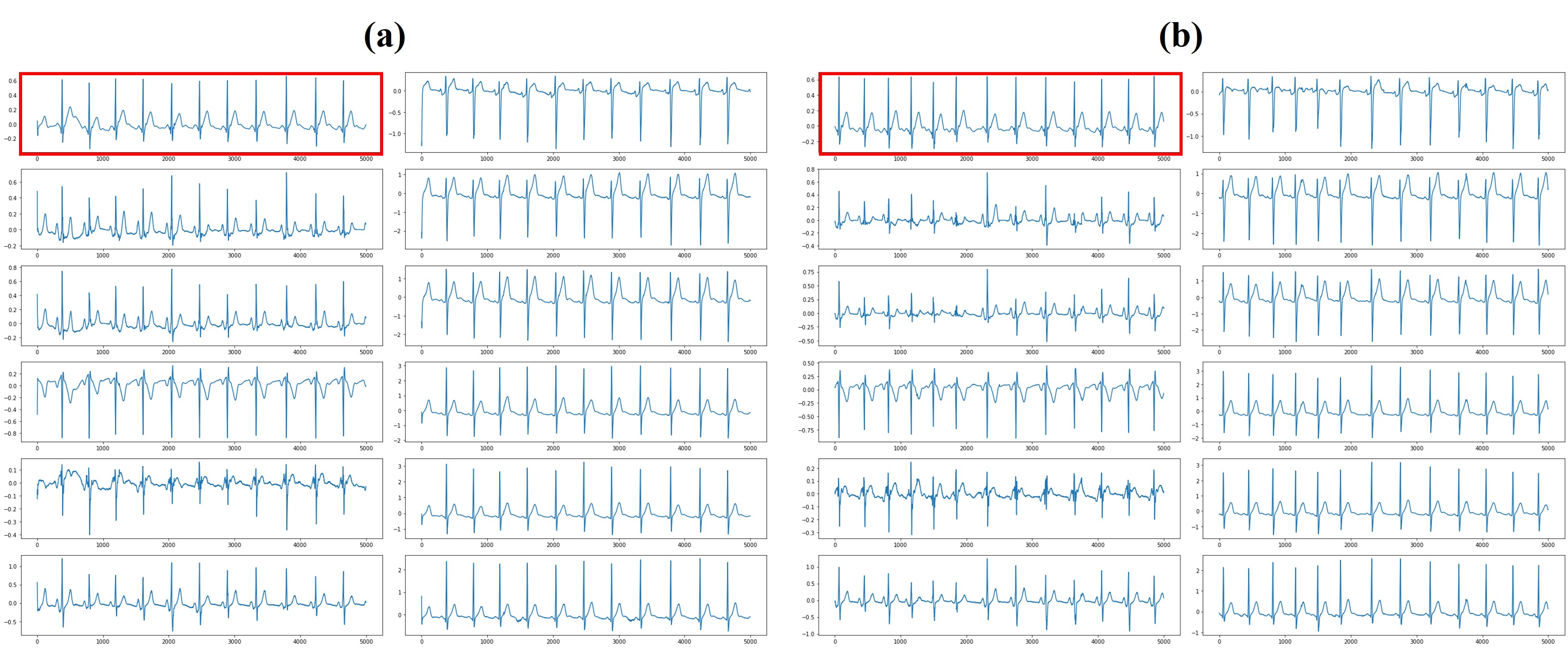


**Supplementary Figure S3 | Generated ECG from apple watch**. (a) and (b) are example of generated ECG signal from apple watch. Red box indicates the original signal obtained by the watch and the rest are generated ECG signal by proposed method. Left column of each figure are limb leads Lead I, Lead II, Lead III, Lead aVR, Lead aVL, Lead aVF and right column is precordial leads from V1 to V6.

The generated ECG were than tested to predict the classification result. Table 2 and Table 3 shows the prediction results. Assuming the recorded ECG from the watch as Lead I, Table 2 was predicted by ResNet model trained by Lead I and Table 3 result is prediction by ResNet model trained by generated 12-Lead. Total 5 measurement and prediction were tested. Since it was measured by apple watch we did not diagnose the ECG. However two results does show significant difference in predicting normal ECG. In future work if database with ECG data measured by watch and standard 12-Lead ECG is available further investigation will be done. Our work shows its possibility that the generated ECG can be used not only in data augmentation methods but also to diagnose with classification models.

Supplementary Table S4

Prediction Result ResNet model Trained with Lead I

|  | Norm | AFIB | RBBB |
| --- | --- | --- | --- |
| Test 1 | 0.999 | 0.002 | 0.001 |
| Test 2 | 0.938 | 0.061 | 0.002 |
| Test 3 | 0.972 | 0.061 | 0.002 |
| Test 4 | 0.993 | 0.008 | 0.001 |
| Test 5 | 0.979 | 0.022 | 0.001 |

Supplementary Table S5

Prediction Result ResNet model Trained with Generated 12-Lead

|  | Norm | AFIB | RBBB |
| --- | --- | --- | --- |
| Test 1 | 1 | 0.001 | 0.001 |
| Test 2 | 0.986 | 0.015 | 0.001 |
| Test 3 | 0.988 | 0.013 | 0.001 |
| Test 4 | 1 | 0.001 | 0.001 |
| Test 5 | 0.96 | 0.04 | 0.001 |

# Generating Without Noise and Baseline Wandering Examples


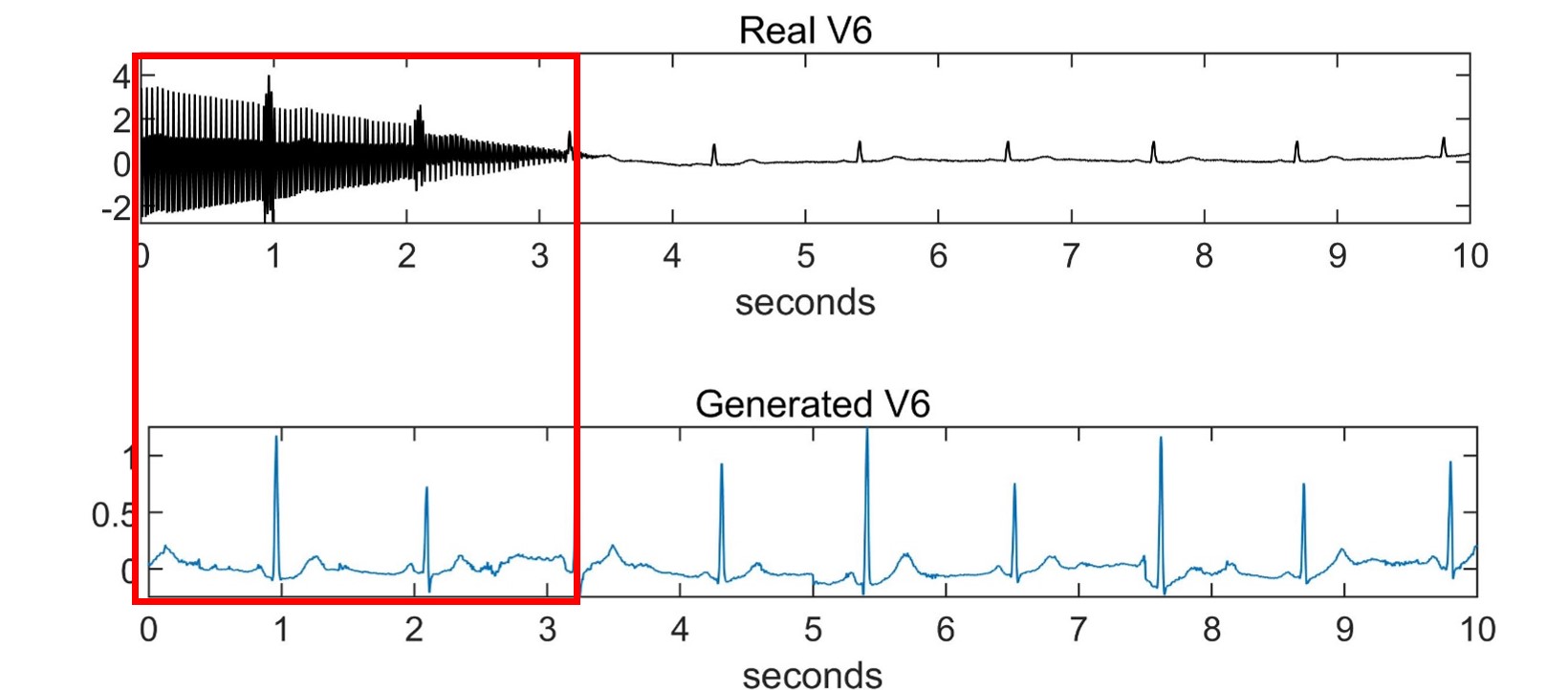


**Supplementary Figure S5 | Missing data example**. Example of distorted or missing data being generated.


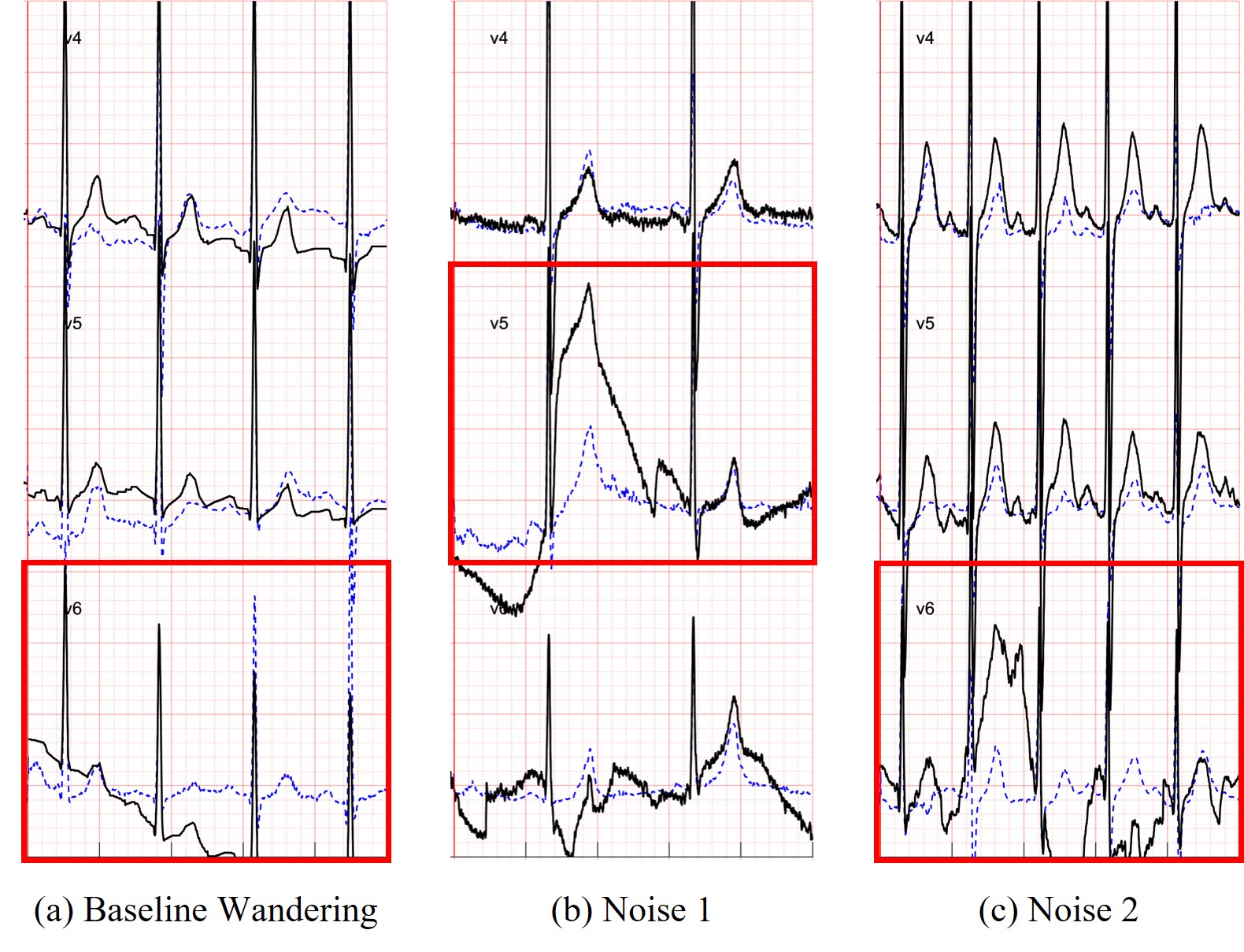


**Supplementary Figure S4 | Baseline and noise removed example**. Blue line is generated ECG and black line is corresponding reference real ECG. (a) is an example of removed baseline wandering. (b) and (c) is an example were noise is removed.

# GAN Model Description


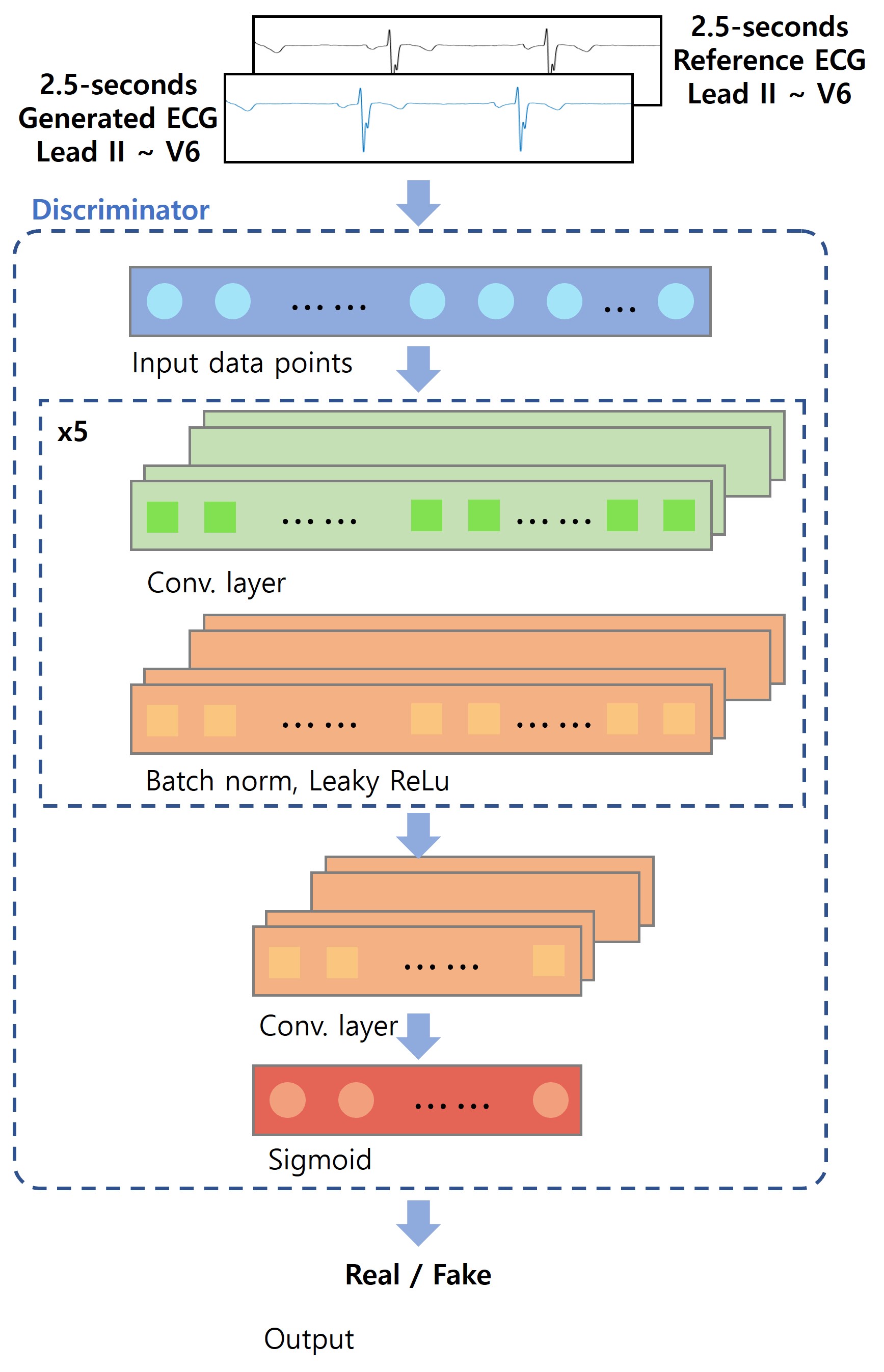


**Supplementary Figure S7 | Flow chart of proposed discriminator**.


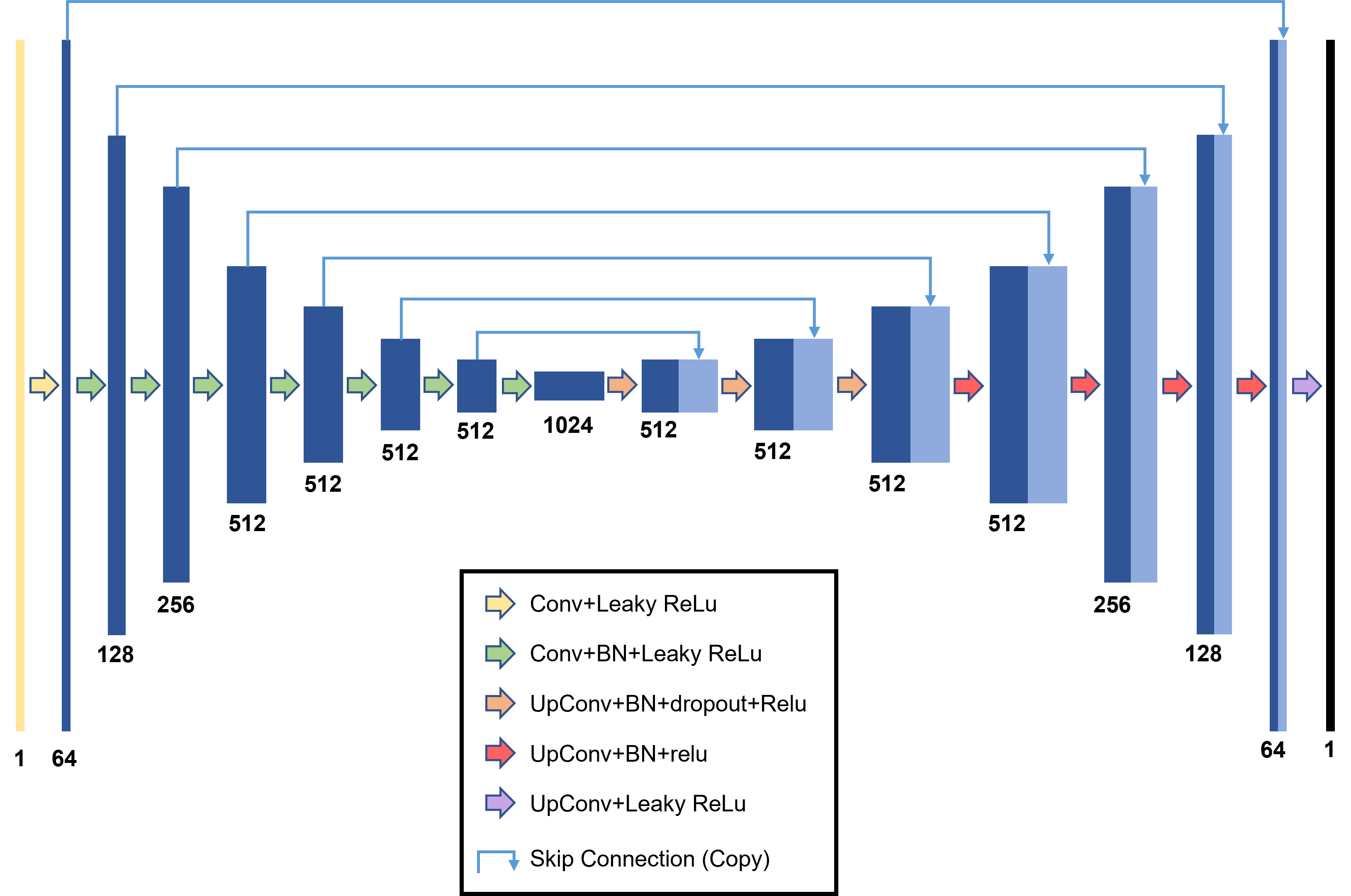


**Supplementary Figure S6 | Flow chart of proposed U-net generator**.
